# Genome-wide analysis of 439 mass spectrometry-based proteomic profiles in a population of 15,035 Scottish individuals

**DOI:** 10.1038/s41467-026-76474-8

**Published:** 2026-09-09

**Authors:** Anne Richmond, Josephine A. Robertson, Hannah M. Smith, Robert F. Hillary, Aleksej Zelezniak, Spyros Vernardis, Aleksandra D. Chybowska, Arturas Grauslys, Jure Mur, Archie Campbell, Camilla Drake, Hannah Grant, Poppy Adkin, Matthew White, Charles Brigden, Christoph B. Messner, David J. Porteous, Caroline Hayward, Andrew M. McIntosh, Daniel L. McCartney, Markus Ralser, Riccardo E. Marioni

**Affiliations:** 1 Institute of Genetics and Cancer, University of Edinburgh, Edinburgh, UK; 2 Molecular Biology of Metabolism Laboratory, The Francis Crick Institute, London, UK; 3 Department of Biology and Biological Engineering, Chalmers University of Technology, Gothenburg, Sweden; 4 Eliptica Limited, The London Cancer Hub, London, UK; 5 Randall Centre for Cell & Molecular Biophysics, King’s College London, New Hunt’s House, London, UK; 6 Department of Psychology, Alzheimer Scotland Dementia Research Centre, University of Edinburgh, Edinburgh, UK; 7 Precision Proteomics Center, Swiss Institute of Allergy and Asthma Research, University of Zurich, Zurich, Switzerland; 8 Institute for Neurocience and Cardiovascular Research, University of Edinburgh, Edinburgh, UK; 9 Department of Biochemistry, Charité Universitätsmedizin Berlin, Berlin, Germany

**Keywords:** Genome-wide association studies, Proteomics, Quantitative trait

## Abstract

Understanding the genetic architecture of the circulating proteome can help determine if a protein is causally linked to disease. Previous large-scale genome-wide association studies of proteins have mostly been conducted on pre-defined, targeted subsets of the proteome, and have often concentrated on low-abundance proteins, many of which don’t exert their main function in serum. Mass spectrometry-based proteomics facilitates the study of high-abundance proteins and their isoforms, focusing on proteins active in blood. In 15035 individuals from Generation Scotland, we perform genome-wide association studies of 439 highly abundant serum protein groups as identified and quantified by liquid chromatography tandem mass spectrometry. We identify 1553 independent SNP signals for 398 proteins (*P* < 1.2×10^−10^). Two-sample Mendelian Randomisation analyses are applied to test if the 398 proteins with significant SNP signals are causally associated with 79 common causes of morbidity and mortality. We report putative causal associations between 13 proteins and 17 outcomes, including neuropsychiatric and cardiovascular conditions. Large-scale genome-wide analyses of the high-abundance proteome complement targeted approaches for the discovery of causal pathways of disease.

## Abstract (web summary)

This study identifies 1,553 significant independent pQTLs for 398 proteins. MR analysis reveals 13 proteins with potential causal associations with 17 disease traits and associations between trypsin inhibitors and neuropsychiatric conditions.

## Introduction

The circulating proteome can capture a readout of lifestyle and environmental exposures and inform disease risk prediction^1^. As a consequence, serum proteins are used as biomarkers in several conditions, such as cardiac Troponin I and T to indicate myocardial infarction^2^, cystatin C to indicate kidney dysfunction^3^, C-reactive protein to indicate inflammation^4^, or prostate-specific antigen as a marker of prostate cancer^5^. In addition to biomarker work, understanding the genetic architecture of blood-based proteins helps to determine causal disease mechanisms and identify drug targets^6^.

To date, large-scale studies have used targeted aptamer- or affinity-based platforms that can assess upwards of 3000 (Olink Explore 3072 panel) and 7000 (SomaLogic SomaScan assay) pre-defined proteins^7–9^. Genome-wide association studies (GWAS) of these proteins have helped to give mechanistic insights into multiple disease outcomes—including those where blood is not the primary tissue of interest^10–13^. However, affinity assay-based techniques have limitations. Quantification of functionally important, high-abundance serum proteins can be inaccurate due to signals exceeding the dynamic range of the array and the presence of different isoforms^14^. They are also limited by a pre-defined list of protein targets, off-target binding and interference caused by autoantibodies^15^, as well as lack of concordance across arrays^16^. In contrast, untargeted approaches for proteomics such as mass spectrometry are unbiased and yield comprehensive and granular coverage of the high-abundance proteome. This non-reliance on an epitope confers higher analytical specificity relative to affinity-based methods and enables direct interrogation of protein isoforms and peptide-level variation. When integrated with genomic data, this resolution facilitates direct mapping of genetic variation to specific peptides and proteins, offering a deeper mechanistic view of genotype-protein relationships. To date, only a small number of studies have performed GWAS on mass spectrometry-measured proteins. However, their sample sizes have been limited to under 3000 individuals^9,17,18^. This is an important limitation as GWAS typically require large sample sizes to achieve adequate statistical power to detect variants with small effect sizes.

Recently, we and others have developed an ultra-high-throughput mass spectrometry proteomic technology that can measure proteins and peptides in excess of 200 samples per day, while advances in sample processing and data analysis have increasingly mitigated the impact of problematic batch effects^19^. The application of this technology to large population biobanks will accelerate insights into the genetic control of the human proteome by providing an unprecedented scale and accuracy of protein quantification^6^.

Here, we characterise the genetic architecture of 439 mass spectrometry-measured proteins in 15,035 individuals from the Generation Scotland study. This study is 5–10 times larger than recently published GWAS of mass spectrometry proteomics^9,17,18^. First, we identify genetic variants that associate with mean differences in serum protein levels (i.e. protein quantitative trait loci, or pQTLs). Second, harnessing these data, we perform Mendelian Randomisation (MR) to explore causal associations between protein levels and 79 leading causes of morbidity and mortality^20,21^. Together, we leverage unbiased protein measurements to provide a catalogue of genetic influences on serum protein levels as well as detail their causal associations with prevalent disease states.

## Results

### pQTL discovery

**Fig. 1:**
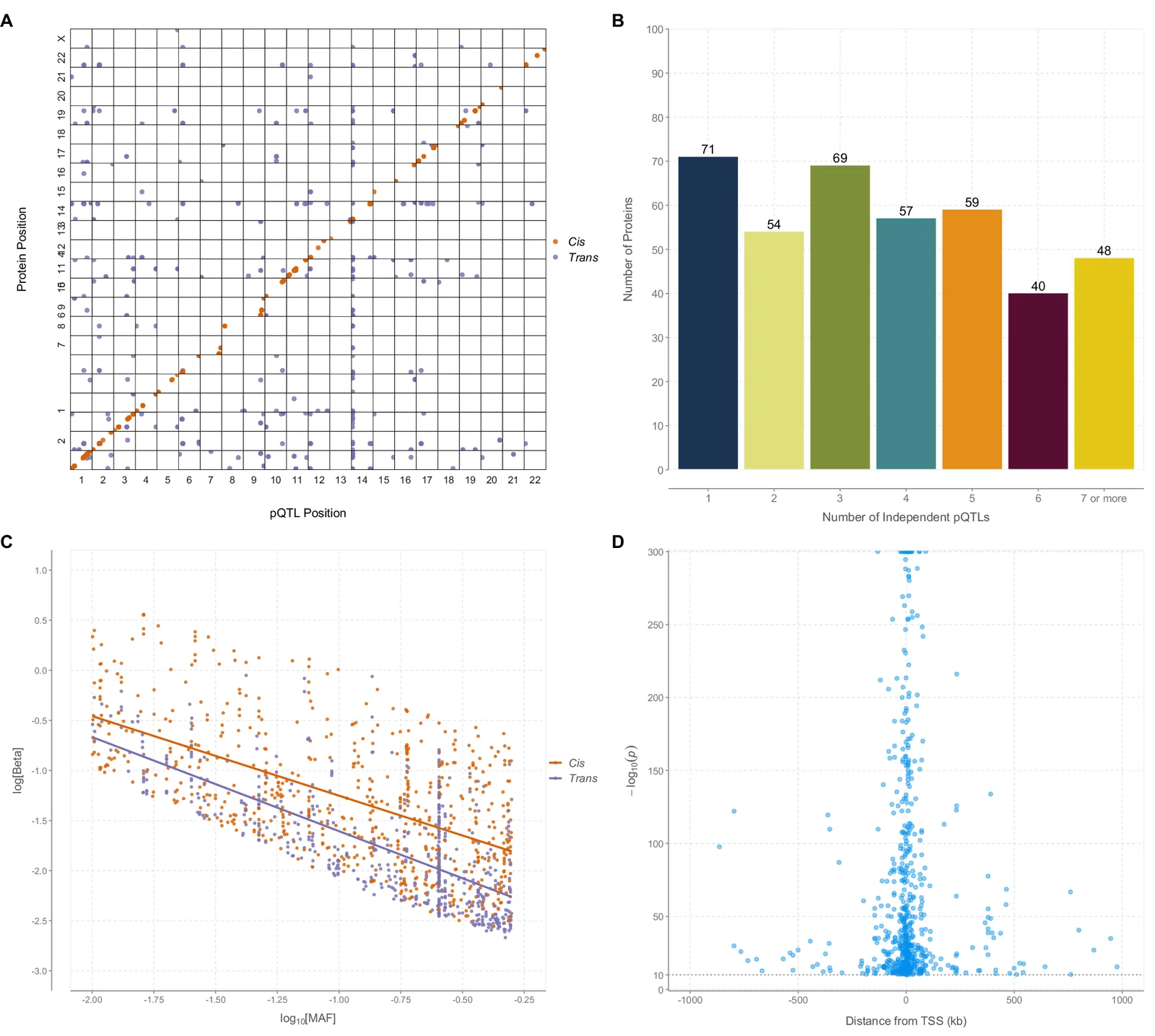
Genome-wide association studies to identify pQTLs for highly abundant proteins as measured by mass spectrometry. **A** The *x*-axis represents the chromosomal location of independent cis and trans pQTLs. The *y*-axis represents the position of the gene encoding the protein. Only uniquely mapped proteins are shown. Cis associations for proteins are marked by orange circles and trans associations (not linked to any protein targets) are marked by purple circles. **B** The number of independent pQTLs per protein (or protein group). **C** Association between the common logarithm of minor allele frequencies and the natural logarithm of absolute effect sizes for cis and trans pQTLs. Only uniquely mapped proteins are shown. Cis (orange circles and line); trans (purple circles and line). **D** Association between the strength of cis pQTL associations and their distance in kilobases (kb) from their respective transcription start sites. pQTL protein quantitative trait locus, kb kilobases. Source data are provided as a Source Data file.

GWAS analyses of 439 serum proteins (mapped to one UniProt ID) or protein groups (mapped to multiple UniProt IDs) levels were conducted using data from 15,035 participants from Generation Scotland. We observed 199,489 genome-wide significant (*P* < 1.2 × 10^−10^) SNP associations with 398 (91%) of the proteins/protein groups. Following conditional analysis, 1553 independent signals were identified with the lead variant defined as the SNP with the lowest *P*-value within the given locus (Supplementary Data 1). 1199 of the significant pQTLs were found in uniquely mapped proteins (i.e, non-protein groups), 656 (54.7%) of which were cis (±1 Mb), and 543 (45.3%) trans (Fig. 1A). Over 40% of discovered pQTLs (404 cis and 200 trans) were associated with a single protein. Our results also indicated the presence of a pQTL hotspot within *SERPINA1*, with 17 independent signals generating 181 associations with 140 proteins. 71 proteins/protein groups had only one independent pQTL whilst 48 protein/protein groups had seven or more (Fig. 1B). When comparing effect size with minor allele frequency, a negative correlation (for cis loci: *r*_Pearson_ = −0.60, *P* = 1.0 × 10^−64^; for trans loci: *r*_Pearson_ = −0.72, *P* = 3.6 × 10^−88^) between the log beta and the log MAF was observed (Fig. 1C). In general, for the same MAF, larger effect sizes were found with cis loci than with trans (Fig. 1C). An inverse association (*r*_Pearson_ = −0.15, *P* = 1.8 × 10^−4^) was found between strength of cis pQTL and distance from transcription start site (Fig. 1D). The pQTL analysis was repeated to include a SNP-sex interaction term to test for sex differences in genetic effects. One significant association (*P* < 1.2 × 10^−10^) was found: a cis locus in Ceruloplasmin (CP) (Supplementary Data 2). This locus remained significant after running a sensitivity analysis which included contraception as a covariate to determine if the signal was being driven by hormonal contraceptive use.

### Replication of known genetic associations

**Fig. 2:**
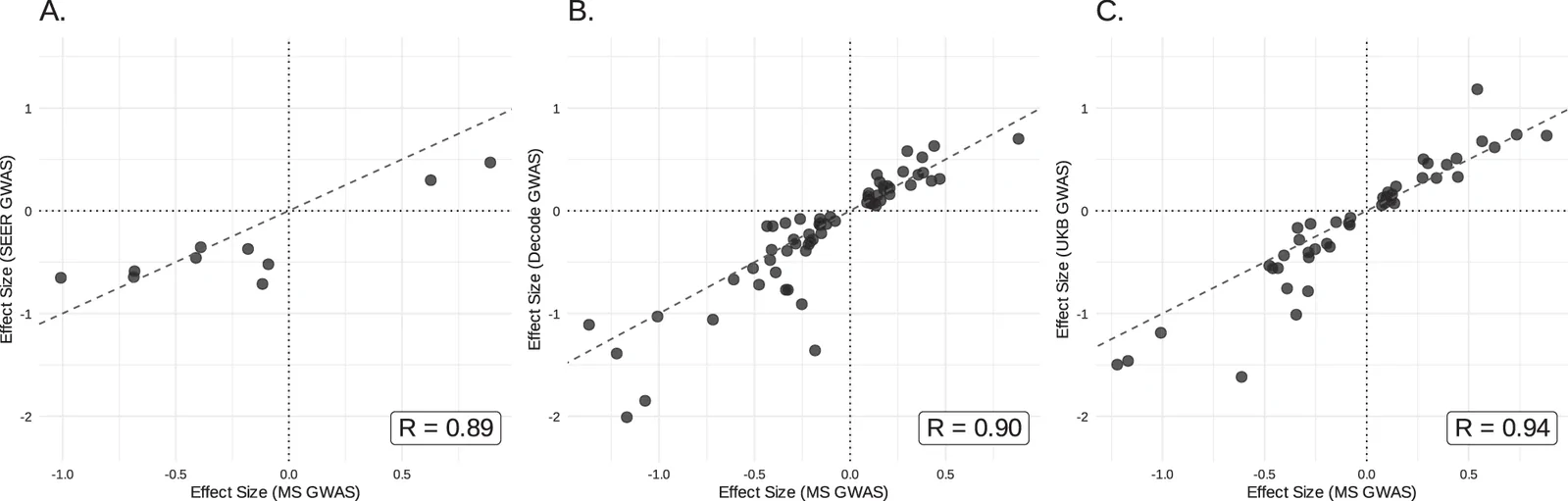
Overlapping associations with comparator pQTL studies. Overlapping associations were defined as those where the genome-wide significant lead variant from the comparator study was within 1 Mb of the discovered lead variant, with a same direction effect size, and has an LD score of >0.6. “MS GWAS” refers to the current study. R denotes Pearson R, calculated using cor.test from the Statistics package in R. **A** 10 overlapping pQTLs with the Seer proteomics GWAS (Suhre et al.^18^); **B** 64 overlapping pQTLs with the Icelandic SomaScan v4 platform GWAS (Eldjarn et al.^10^); **C** 46 overlapping pQTLs with the UK Biobank GWAS (Sun et al.^11^). Source data are provided as a Source Data file.

The significant pQTLs discovered in our study were compared to three previous proteomics GWAS. These include two large-scale affinity-based proteomics GWAS using Olink Explore 3072 PEA in the UK Biobank^11^ and SomaScan v4 in the deCODE study^10^, and a smaller-scale mass-spectrometry proteomics GWAS using the MS-based Seer Proteograph platform in the Tarkin study^18^. For all significant pQTLs in the current study, we performed lookup analyses in these studies, where the same protein was assessed and also demonstrated significant associations. We replicated 46 of 199 (23.12%), 64 of 275 (23.27%) and 10 of 25 (40%) pQTLs with the Sun et al.^11^, Eldjarn et al.^10^, and Suhre et al.^18^, studies, respectively (see Table 1). Effect sizes of the overlapping associations correlated highly between platforms (*R*_Pearson_ = 0.94 (MS:UKB^11^)); 0.90 (MS:deCODE cohort^10^); 0.89 (MS:Tarkin^18^) (Fig. 2). A list of the overlapping proteins-SNP associations can be found in Supplementary Data 3, 4 and 5.

**Table 1:** Comparison of discovery pQTLs with three comparator studies

Comparator GWAS study	Sun et al.^11^	Eldjarn et al.^10^	Suhre et al.^18^
Cohort	UK Biobank	Decode	Tarkin
Sample size	54,219	35,892	1260
Proteomics platform	Olink	SomaScan	Seer
N proteins tested	2923	4719	1980
Overlap of proteins*	58	75	114
Overlap of proteins with significant associations*	51	69	22
N significant associations with overlapping proteins in comparator study	199	275	25
Replicated pQTLs (%)*	46 (23.1%)	64 (23.3%)	10 (40%)

The three comparator studies considered include two large-scale affinity-based proteomics GWAS using Olink Explore 3072 PEA in the UK Biobank^11^ and SomaScan v4 in the deCODE study^10^, and a smaller scale mass-spectrometry proteomics GWAS using the MS-based Seer Proteograph platform in the Tarkin study^18^.

*These rows refer to overlap between our study (Eliptica MS GWAS) and the respective study for each column.

### MR of protein levels and 79 disease and health outcomes

**Fig. 3:**
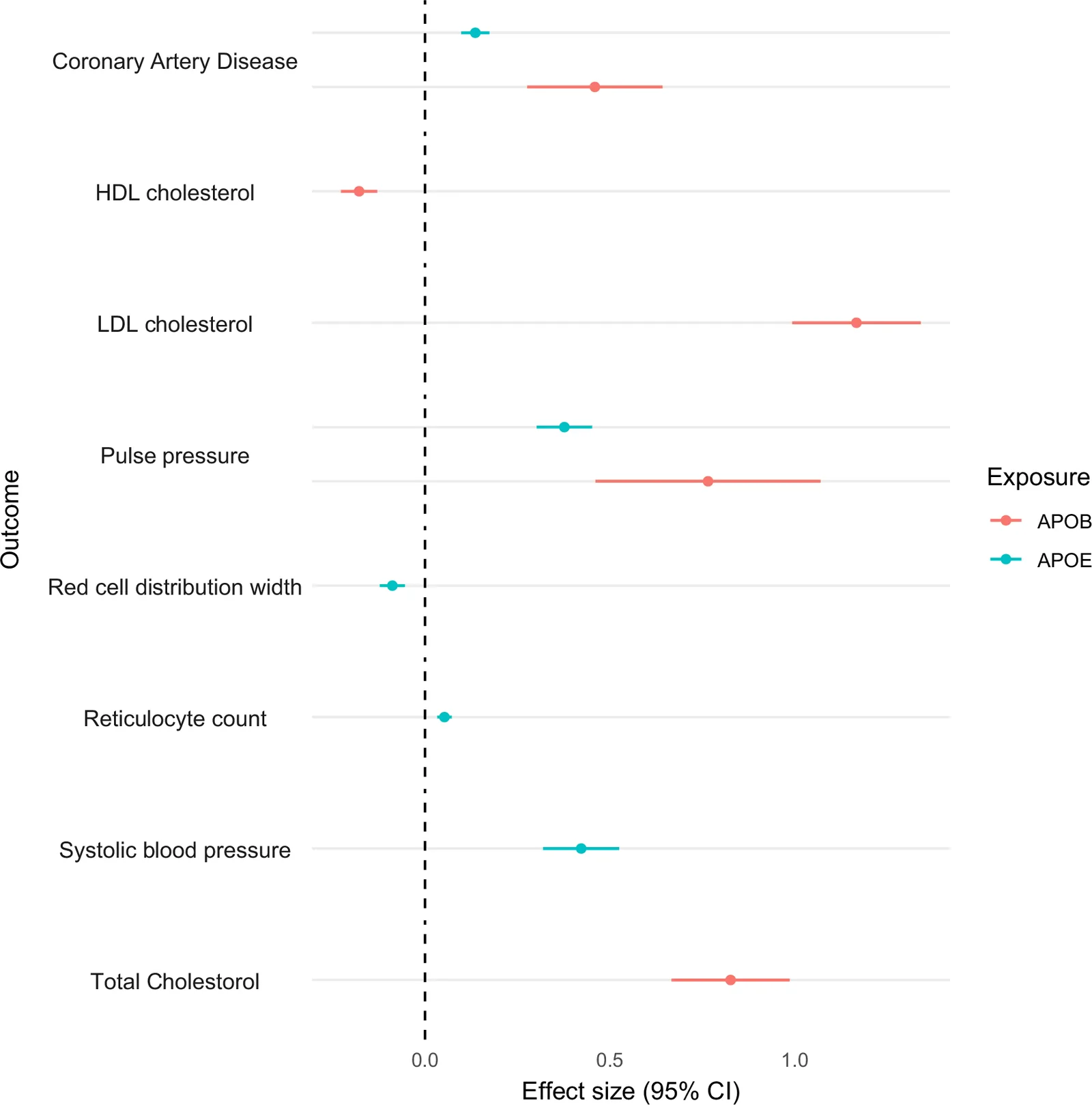
Traits associated with apolipoprotein B (APOB) apolipoprotein E (APOE) in MR analysis. Data shown are effect sizes and 95% confidence intervals of the significant (*P* < 1.59 × 10^−6^) causal associations of APOB and APOE with 8 traits following MR analysis. Effect sizes displayed are log odds for binary traits and beta values for continuous traits, per SD of the rank transformed protein.Where multiple isoforms of the same protein were associated with one trait, the association with the largest effect size is shown. APOB and APOE: *N* = 15,035. Coronary Artery Disease: *N* = 184,305. HDL cholesterol, LDL cholesterol and Total cholesterol: *N* = 188,577. Pulse pressure and Systolic blood pressure: *N* = 757,601. Red cell distribution width and Reticulocyte count: *N* = 173,480. Source data are provided as a Source Data file.

**Fig. 4:**
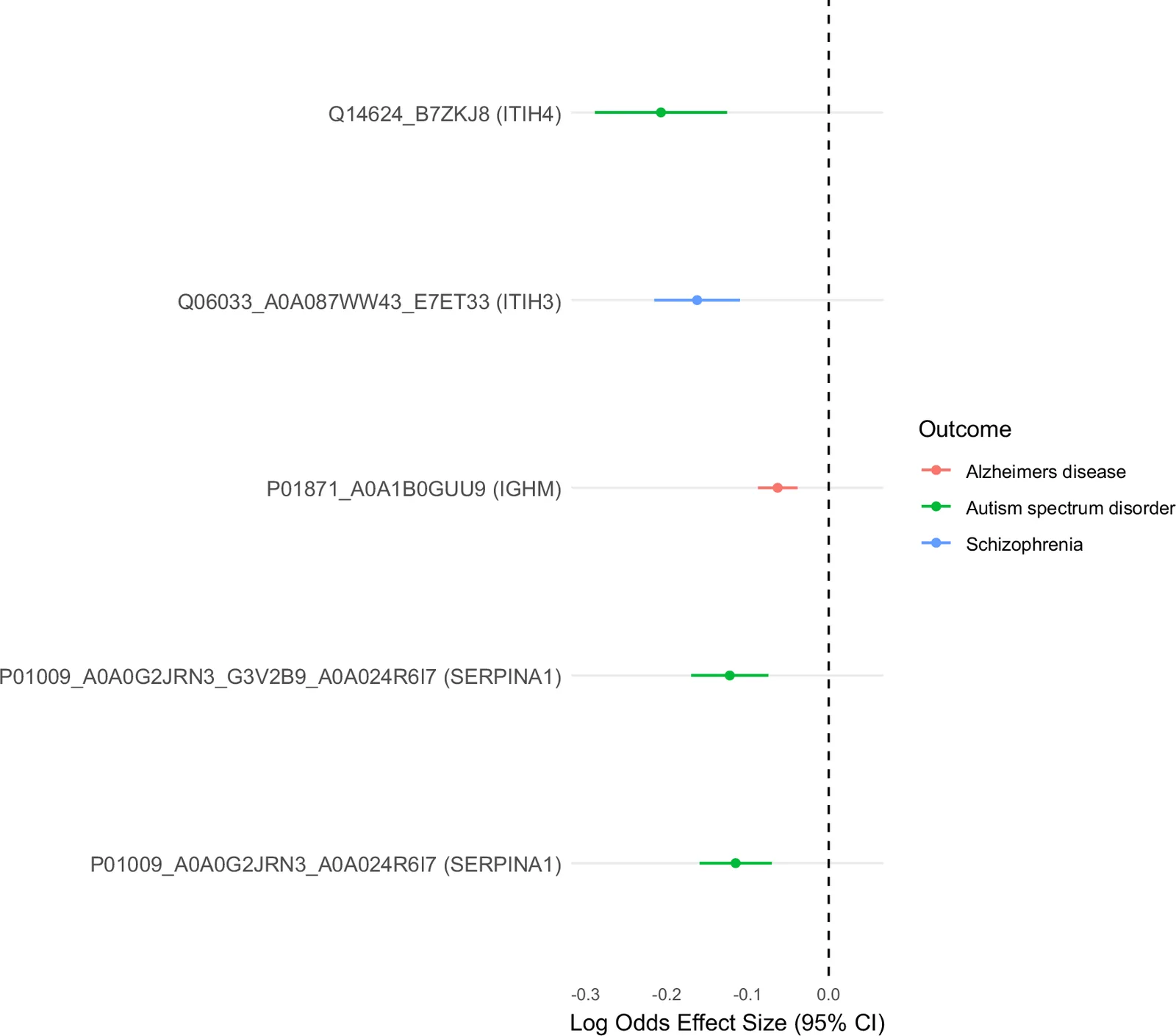
Proteins causally associated with Alzheimer’s disease (AD), Autism Spectrum Disorder (ASD) and Schizophrenia in MR analysis. Data shown are log odds effect sizes and 95% confidence intervals of the significantly (*P* < 1.59 × 10^−6^) associated proteins with the neuropsychiatric conditions AD, ASD and Schizophrenia following MR analysis. Effect sizes are per SD of the rank transformed proteins. ITIH4, ITIH3, IGHM and SERPINA1: *N* = 15,035. Alzheimer’s disease: *N* = 455,258. Autism spectrum disorder: *N* = 46,350. Schizophrenia: *N* = 82,315. Source data are provided as a Source Data file.

Identifying causal protein—disease pathways could aid the discovery of novel drug targets; human genetic evidence increases the success rate for approval of developed drugs^22,23^. Given this, we tested whether any of the proteins or protein groups were causally associated with 79 disease or health outcomes (for full list see Supplementary Data 6). We identified 13 proteins that were putatively causal factors for 17 traits (Supplementary Data 7), giving a total of 31 significant results (inverse variance weighted (IVW) method with *P* < 1.59 × 10^−6^, at least one other method with *P* < 0.05 and a minimum of 3 SNP instruments). The causal direction was from exposure (protein) to disease outcome, as determined by the Steiger directionality test. The proteins apolipoprotein E (APOE) and apolipoprotein B (APOB) were each associated with five of the cardiovascular-related and metabolic traits (Fig. 3). The strongest APOE association was with systolic blood pressure (IVW beta = 0.42, *P* = 8.5 × 10^−16^) while the strongest APOB association was with LDL cholesterol (IVW beta = 1.17, *P* = 1.4 × 10^−39^). This was supported by posterior probabilities of >0.99 (H4) for both LDL and HDL cholesterol in colocalization analysis, suggesting a shared causal variant. Significant MR results were also found for three neuropsychiatric traits: autism spectrum disorder (ASD), schizophrenia, and Alzheimer’s disease (AD). The trypsin inhibitors inter-alpha-trypsin inhibitor heavy chain 3 (ITIH3) and inter-alpha-trypsin inhibitor heavy chain 4 (ITIH4) were associated with Schizophrenia (IVW log odds = −0.16, *P* = 1.7 × 10^−9^) and ASD (IVW log odds = −0.21, *P* = 6.3 × 10^−7^), respectively. Alpha-1-Antitrypsin (AAT) was also found to be associated with ASD (IVW log odds = −0.11, *P* = 4.2 × 10^−7^). AAT, ITIH3 and ITIH4 were also found to have associations or interactions with each other as defined by StringDB^24^ (Supplementary Fig. 1). One protein, Immunoglobulin heavy constant mu (IGHM), showed evidence for a causal association with Alzheimer’s Disease (IVW log odds = −0.063, *P* = 4.3 × 10^−7^) (Fig. 4). To further support our findings of causal associations between our protein exposures and disease outcomes we ran colocalisation analysis using coloc. 15 out of the 31 (48.4%) significant MR results showed strong evidence (PP.H4.abf>0.8), with a further two (6.5%) showing moderate evidence (PP.H4.abf>0.6) of a shared causal variant between the exposure and outcome (Supplementary Data 7).

### Gene set and tissue enrichment analysis

To further understand the biological mechanisms underlying the QTL signals for each protein with a significant MR result, we ran gene set enrichment analysis using FUMA. The full list of gene set databases used to test for enrichment can be found in the methods. Protein-specific QTL gene sets were tested for enrichment; significant findings were observed for all 17 proteins under investigation (*P* < 0.05, Supplementary Data 8). Pathways related to lipids and lipoproteins saw the most enrichment, with 144 pathways from 10 gene set databases enriched across 11 of the proteins. Numerous disease-related pathways from the GWAS Catalog geneset database were significantly enriched. Pathways related to schizophrenia and age-related macular degeneration (AMD) were the most prominent, showing enrichment across 7 proteins, followed by Alzheimer’s disease and ASD, whose associated pathways were enriched across 5 proteins.

Genes associated with ITIH4 and ITIH3, two of our proteins with significant MR results with psychiatric conditions, were significantly enriched in pathways related to the psychiatric conditions ASD, Schizophrenia, Bipolar Disorder and Depression. They were also both significantly enriched in brain-related pathways including brain morphology, subcortical volume and cortical surface area. Genes associated with IGHM and APOM, proteins with MR associations with Alzheimer’s disease and neutrophil count, respectively, were both significantly enriched in immunology-related pathways, including interferon signalling, autoimmune thyroid disease and MHC class II protein complex pathways.

Nine proteins were significantly overrepresented in at least one tissue type (*P* < 0.05, Supplementary Data 9). Genes associated with IGHM were found to be significantly overrepresented in blood, lung and spleen tissue and underrepresented in ovarian tissue and genes associated with SERPINA1, a protein with an MR association with ASD, were found to be overrepresented in liver tissue.

## Discussion

Here, we describe a large-scale GWAS of 439 serum protein/protein group levels from the highly-abundant proteome fraction in the Generation Scotland cohort (*N*_samples_ = 15,035). The high-abundance proteome fraction is important for human physiology as it contains proteins important for metabolism, nutrient transport and the innate immune system, including complement and coagulation factors. However, these proteins can be challenging to quantify as they exist in many isoforms and can saturate the measurement reagents. We identified 1553 significant (*P* < 1.2 × 10^−10^) independent pQTLs with 398 of the proteins/protein groups. Of the 1199 loci significantly associated with unique proteins, we reported 656 as cis and 543 as trans.

We identified pQTLs that replicate those in previous proteomics GWAS, with highly correlated effect sizes (*R*_Pearson_ > 0.89). We replicate approximately 23% of associations within overlapping proteins with significant associations identified in two large-scale affinity-based studies using the SomaScan v4 and Olink platforms (sample sizes ~ 35,892 and 54,219, respectively). We additionally compared our results with a smaller-scale mass-spectrometry proteomics GWAS using the Seer platform (*N*_samples_ = 1260), replicating 40% of the identified associations within overlapping proteins with significant associations in both studies. This degree of replication with the affinity-based studies is higher than that of this previous MS proteomics study^18^. Our cross-platform replication demonstrates the validity of a mass-spectrometry approach and further helps to identify pQTLs that are unlikely to be driven by antibody cross-reactivity or epitope-binding artifacts.

Our results identify a trans hotspot within *SERPINA1*, with at least one significant association found with 140 of the 439 proteins. *SERPINA1* encodes a serine protease inhibitor, AAT, predominantly produced in the liver^25^. Accordingly, significant GWAS loci associated with AAT were found to be overrepresented in liver tissue on tissue-enrichment analysis. AAT is involved in many biological processes, including inflammation, coagulation, homeostasis and the immune response^26^. Mutations in *SERPINA1* can result in AAT deficiency, increasing risk of liver and lung disease^25,27^. Given its involvement in a diverse range of pathways, it is biologically plausible that pQTLs within *SERPINA1* have wide-ranging associations.

Whilst research into biomarkers for disease has identified many protein – disease associations, unpicking the biological mechanisms or causality for these associations is more challenging. MR analysis offers one route to assess for putative causal relationships between associations. We used two-sample MR to investigate causal associations between any of the proteins with identified pQTLs and 79 disease/health outcomes. There was evidence for causal associations between 13 proteins and 17 traits, including several neuropsychiatric disorders and cardiovascular-related traits.

MR analysis identified causal associations between APOB and APOE and coronary artery disease (CAD) and pulse pressure. Circulating APOB concentration is considered a biomarker for atherogenic lipids that are known to promote fatty deposits in arteries^28^. Polymorphisms in the *APOE* e4 allele have been shown to increase the risk of CAD^29^. Gene set enrichment analysis of APOE-associated loci demonstrates enrichment in metabolic pathways and, via the GWAS Catalog geneset database, diseases such as Alzheimer’s Disease, non-alcoholic fatty liver disease and metabolic disorders.

MR also revealed putative causal associations between anti-inflammatory proteins and neuropsychiatric disorders. Evidence is building for a role of immune dysregulation in the aetiology of ASD and schizophrenia^30,31^. In our analysis, AAT and ITIH4 associated with ASD, and ITIH3 associated with schizophrenia, with all associations suggesting that increased protein levels reduce the odds ratio of diagnosis. These proteins have both anti-inflammatory and immunomodulatory functions^32^, have been implicated in the pathogenesis of autoimmune conditions and have also been proposed as potential therapeutic tools to manage these conditions^33^. They are produced both peripherally and in nervous-system tissue^24^. Further, gene set enrichment analysis for the significant GWAS loci in ITIH4 and ITIH3 identified significant enrichment in pathways related to psychiatric disorders, including ASD, Schizophrenia, Bipolar Disorder and Depression. This also identified significant enrichment in pathways related to brain morphology, subcortical volume and cortical surface area, aligning with well-described structural brain changes in Schizophrenia^34^ and ASD^35^. Results for neither protein demonstrated tissue-specific enrichment. Our results build on existing evidence of a role of inter-alpha-trypsins in neuropsychiatric disorders from both genetic and proteomic studies. Schizophrenia is a highly heritable condition^36^ and previous large-scale GWAS studies have identified SNPs in *ITIH3/4* in association with Schizophrenia^37–39^ and also between *SERPINA1* and ASD^40^ and *ITIH3/4* and ASD^41^. However, colocalisation analysis (PP(H3) > 0.99) did not support the same causal variant for ITIH3 and Schizophrenia, ITIH4 and ASD or AAT and ASD.

A few small-scale studies have identified associations between AAT and ASD. One study, using mass spectrometry proteomics, identified increased expression levels of AAT in participants with ASD (*N*_cases_ = 30, *N*_controls_ = 30)^42^; similarly, another study using mass spectrometry proteomics (*N*_cases_ = 30, *N*_controls_ = 30) identified increased AAT in children with ASD^43^. However, although our results suggest that increased AAT could be protective against ASD, colocalisation analysis revealed a posterior probability (PP) of H3 as >0.99, implying no shared causal variant. Associations between AAT and Schizophrenia have also been demonstrated^44^, though our analysis did not identify a significant causal association. Our results support the hypothesis that AAT may perform an immunomodulatory role, which is protective against ASD. However, given that we have identified *SERPINA1* as a trans-hotspot for associations with multiple other proteins, including overlap in the SNP associations (Supplementary Data 3 and Supplementary Data 4), it is possible that genetic variants may affect the disease outcome through alternate pathways despite a non-zero Egger MR regression intercept.

Interestingly, we did not identify significant associations with Schizophrenia for all protein or protein groups mapped to AAT (total in the dataset = 5), though effect directions were concordant. These different entries may reflect different isoforms of the protein, which is known to have complex regulation and post-translational modifications^33^. Our findings of SERPINA1 as a trans pQTL hotspot further reinforce the complexity of its involvement in diverse molecular pathways. Mass spectrometry proteomics may prove to be a useful tool for studying similarly complex proteins, aiding the identification and study of different isoforms.

We also identified an MR-significant association between immunoglobulin heavy constant mu (IGHM), and Alzheimer’s disease (AD). IGHM is a component of immunoglobulin M (IgM), an antibody involved in the adaptive immune response and a function of which is to target and clear amyloid beta deposits. Gene set enrichment analysis for the significant GWAS loci associated with this protein identified enrichment in pathways related to interferon signalling, increasingly implicated in Alzheimer's disease^45^, and also pathways related to autoimmune thyroid disease and MHC class II protein complexes. Further, the IGHM-associated genes were significantly enriched in blood, lung and spleen, aligning with immunological functions. The immune system is thought to play a role in the progression of AD^46,47^ and decreased levels of IgM have been found in patients with AD than with age and sex matched healthy controls (*n*_cases_ = 26, *n*_controls_ = 26; *n*_cases_ = 30, *n*_controls_ = 30)^48,49^. However, findings on this topic have been inconsistent as IGHM has previously been shown to be a potential biomarker for AD, with increased levels found in patients with AD than with healthy controls (*n*_cases_ = 45, *n*_controls_ = 45)^50^. IgM levels in frontal cortex tissue have also been found to be increased in people with late-stage (stages V and VI) AD compared to healthy controls of the same age (*n*_cases_ = 11, *n*_controls_ = 9)^50,51^. Further, colocalisation analysis revealed a posterior probability >0.99 for H3, suggesting that while both the disease and protein have causal variants in the region, they do not share a causal variant.

Technological advances in mass spectrometry are increasing its potential for application both in large cohorts and clinical settings^52^. Its untargeted approach avoids bias associated with antibody- or aptamer-based protein panels. Further, mass spectrometry best captures the high-abundance serum proteins^15^, which are not well quantified by assays reliant on an epitope, due to saturation and the presence of multiple isoforms^14,53^. Isoforms that modify protein affinity to assay epitopes can interfere with MR analyses dependent on these results. Mass spectrometry is robust to isoform effects and therefore an important technique for identification or verification of disease-relevant pQTLs^54^, in addition to allowing exploration of high-abundance proteins less well captured by assay-based methods^55^. These high-abundance proteins are typically involved in processes occurring within the blood, such as nutrient transport, coagulation and innate immunity and have been less prioritised in proteomic biomarker work thus far^56^. By concentrating on the highly abundant serum proteome fraction, we have not investigated lower-abundance proteins, predominantly associated with cell signalling or leakage from tissues^57^, which can yield meaningful biological insights^58^.

This study benefits from a large sample size (*N* = 15,035) for pQTL analyses, giving us the ability to detect small effects. Our replication analysis, which included comparison with three independent studies using three different proteomics technology platforms, demonstrated 23–40% replication. Nonetheless, we acknowledge that replication in an external cohort with proteomic data generated using the same methodology would be desirable. Additionally, our study and comparative analysis included individuals of European ancestry only. Future work should focus on generating mass spectrometry datasets and replicating findings in non-European cohorts. This study provides a valuable pQTL resource and suggests potential causal relationships between proteins and health conditions. Follow-up studies will be required to determine the underlying mechanisms of these relationships and to confirm their validity.

We used a recently developed high-throughput mass spectrometry platform for a large-scale GWAS study of high-abundant circulating proteins, many of which function in plasma. Following pQTL identification, conditional and joint analysis revealed 1553 significant and independent pQTLs for 398 proteins or protein groups, including a trans pQTL hotspot in *SERPINA1*. MR analysis revealed 13 proteins that have potential causal associations with 17 disease traits, though the presence of a shared causal variant was only supported by subsequent colocalisation analysis for 17 of the 31 significant MR results. Within these results, we found associations between trypsin inhibitors and the neuropsychiatric conditions ASD and schizophrenia. This demonstrates that the combined approaches of GWAS and MR can reveal biologically relevant protein networks which warrant further investigation for understanding of disease.

## Methods

### Ethics

All components of Generation Scotland received ethical approval from the NHS Tayside Committee on Medical Research Ethics [REC Reference Number: 05/S1401/89]. Generation Scotland has also been granted Research Tissue Bank status by the East of Scotland Research Ethics Service [REC Reference Number: 20-ES-0021], providing generic ethical approval for a wide range of uses within medical research. All participants provided written informed consent. This study was performed in accordance with the Helsinki Declaration.

### Generation Scotland

Generation Scotland is a family-based cohort study of 23,960 volunteers, aged 18–99 at recruitment^59^. Recruitment occurred between 2006 and 2011 when volunteers attended a baseline clinic where they completed health and lifestyle questionnaires, provided samples for biochemistry and omics analyses, and gave consent for data linkage to their medical records. There were 5573 families with a mean size of 4 members and 1400 participants without relatives. The median age at baseline was 47 years, and the sample was 59% female. In the 15,035 individuals with both genetic and protein data, the median age (range of 18–99 years) was 49 years, and the sample was 58% female.

Sample sizes for analysis were determined by the size of the cohort. Exclusions were made based on missingness in proteomic and covariate data and quality control of genetic data. The demographics of the cohort can be observed in Supplementary Data 10.

### DNA extraction and genotyping

Samples were collected, processed and stored using standard operating procedures previously reported^60,61^ and managed through a laboratory information management system at the Edinburgh Clinical Research Facility. A Nucleon Kit (Tepnel Life Science) with the BACC3 protocol was used for DNA extraction. DNA concentrations were determined using Picogreen and diluted to 50 ng/µL for genotyping.

Genotyping was carried out using one of two arrays - the Illumina HumanOmniExpressExome-8v1-2_A or HumanOmniExpressExome-8v1_A. Quality control steps^61^ included filtering for single-nucleotide polymorphisms (SNPs) with missingness (>2% across all individuals), minor allele frequency (MAF) > 1% with Hardy–Weinberg *p*-value < 1 × 10^−6^. Samples were removed based on sex mismatches with the phenotypic database and on missingness (>2% across SNPs). Genetic outliers were identified as lying more than six standard deviations (SDs) beyond the mean on the first two principal components upon integration with the 1000 Genomes population^62^. These individuals were removed, leaving a dataset of 604,858 SNPs and 20,032 individuals^63^. SNP data were then imputed using the Haplotype Reference Consortium (HRC) imputation panel^64^, yielding 24,161,581 variants with MAF > 1% and INFO > 0.4 for the downstream analyses.

### Proteomics

High-throughput mass spectrometry proteomics was carried out using our in-house developed method on serum samples pre-processed for protein denaturation and trypsinisation^65,66^.

Serum samples (5 μl) were denatured in a solution of 50 μL of 8 M urea (Sigma-Aldrich, 1084870500) and 0.1 M ammonium bicarbonate (ABC, Thermo Fisher, 15645440) at pH 8.0. 5 μL of 50 mM dithiothreitol (DTT, Sigma-Aldrich, 43815) was used to reduce the proteins for 1 h at 30 °C. The proteins were then alkylated in the dark for 30 min using 5 μL of 100 mM iodoacetamide (IAA, Sigma-Aldrich, I1149). Dilution of the sample was performed with 340 μL of 0.1 M ammonium bicarbonate to a concentration of 1.5 M urea. 200 μL of the solution was treated with trypsin (12.5 μL, 0.1 μg/μL, Promega, Sequence Grade, V5117) overnight at 37 °C (at a 1/40 trypsin/total protein ratio) to digest the proteins. 25 μL of 0.1% v/v formic acid (FA, Thermo Fisher Scientific, 85178) was used to quench the digestion. C18 96-well plates (BioPureSPN Macro 96-well, 100 mg PROTO 300 C18, HNS S18V-L) were used to purify the peptides, which were eluted with 50% v/v acetonitrile (ACN, Thermo Fisher, 10001334), dried by a vacuum concentrator (Eppendorf Concentrator Plus), and redissolved in 50 μL of 0.1% v/v formic acid.

Liquid-chromatography-mass spectrometry (LC-MS) was carried out on the processed samples using the Agilent 1290 Infinity II system (LC) and a Triple TOF 6600 mass spectrometer (SCIEX)^19^. A Phenomenex Luna Omega LC Column (1.6u PS C18 100A, 30 × 2.1 mm) was used to separate peptides of a 2 μg total over a 3-min gradient. Buffer A (0.1% v/v FA) and Buffer B (ACN/0.1% v/v FA) were used to create a linear gradient, starting from 1% Buffer B and reaching 40% Buffer B over 3 min with an 800 μL/min flow rate. At a 40% gradient, washing and re-equilibration steps were carried out in the following manner: Buffer B was increased from 40% to 80% over 0.5 min, followed by 80% Buffer B for 0.2 min, and then decreased from 80% to 3% Buffer B over 0.1 min. Re-equilibration at 3% Buffer B was maintained for 1 min until the next sample injection.

Sample acquisition was carried out using a Scanning SWATH method^19^. High-sensitivity mode was used for the mass spectrometer. The source conditions were as follows: source gas one at 15 psi, source gas two at 20 psi, curtain gas at 25 psi, temperature at 0 °C, IonSpray floating voltage at 5500 V, and declustering potential at 80 V. Rolling collision energies were determined using the following equation: CE = 0.034×*m/z*+2, where *m/z* represents the centre of the scanning quadrupole bin. The Scanning SWATH parameters were as follows: total cycle time 0.69 s, transmission window width 10 Da, Sample duration 3.103 min, precursor range 450–850 Da, fragment range 100–1500 Da and effective accumulation time 16.90 ms.

DIA-NN (version 1.8.12)^67^ in Robust LC (high precision) was used to process the raw data using default parameters. A human plasma project-independent public spectral library^68^ was used for identification and refined with parameters estimated from the data being processed^65^. Precursor false discovery rate (FDR) filtering was set to 1%. Additional processing was carried out in R. A gene group *q*-value filter of 1% was applied. Samples with a minimum precursor identification of 2000 were retained in the analysis, and an 80% prevalence rate for precursors was used to filter QC samples. A cubic-spline model was fit using the repeat injections of pooled QC samples and used to correct samples for within-batch drift^69^. Between-batch correction was performed using the limma v3.54.2 linear batch-correction algorithm^70^.

439 proteins and protein groups were considered; 133 (30.3%) of these were uniquely mapped to one UniProt ID (termed “proteins”), with the remaining 306 (69.7%) mapping to multiple potential UniProt IDs (termed “protein groups”). 199 of these protein groups represent different isoforms of the same protein, and 107 mixed protein groups where the signal mapped to different proteins. 138 out of the 199 isoform signals were mapped to proteins that were identified more than once (repeated isoform groups). We use a “GX” naming system, where “G” signifies a group and “X” refers to a number to identify these repeated isoform groups (Table 2). For example, Apolipoprotein D was identified twice, with one of the signals annotating to three isoforms [UniProt identifiers: P05090, C9JF17, C9JX71] and was given the name Apolipoprotein D (G1) and another signal mapped to two isoforms [UniProt identifiers: P05090.C9JF17], therefore was named Apolipoprotein D (G2). 61 signals were annotated to protein isoform groups that did not repeat; these therefore only have a “G” after their protein name. We used a “PG X” naming system to identify the 107 signals that map to multiple different proteins. For example, one signal is annotated to multiple immunoglobulins (Immunoglobulin lambda variable 3-27, Immunoglobulin lambda variable 3-25 and Immunoglobulin lambda variable 3-16), and therefore is referred to as protein group 1 (PG 1). As can be observed with the PG1 example, in many cases, the protein groups with mixed labels contain related proteins. In some instances, a protein is identified as a unique signal and as several protein groups. For example, one mass spectrometry signal mapped to Alpha-1-antitrypsin identified via a single UniProt ID P01009; therefore, it was named Alpha-1-antitrypsin. However, five other mass spectrometry signals mapped to five different combinations of UniProt IDs that were all related to Alpha-1-antitrypsin and were therefore named Alpha-1-antitrypsin G1 to G5. Given that these groups originate from different signals, they were maintained as separate entities in downstream analysis. Annotation of the 439 proteins considered in this study is provided in Supplementary Data 11.

**Table 2:** Illustration of protein annotation strategy

Protein category	Count (*n*)	Example UniProtID(s)	Gene symbol(s)	Final annotation
Unique Proteins	133	P00450	CP	Ceruloplasmin
Gene-Derived Protein Groups (G)	199	P02647.F8W696	APOA1, APOA1	Apolipoprotein A-I (G)
		P00736.F5H2D0.B4DPQ0	C1R, C1R, C1R	Complement C1r subcomponent (G1)
		P00736.F5H1V0.F5H6Y3.B4DPQ0	C1R, C1R, C1R, C1R	Complement C1r subcomponent (G2)
Mixed Protein Groups (PG)	107	P80748.A0A075B6K5	IGLV3-21, IGLV3-9	PG 70 (IGLV3-21, IGLV3-9)
		P00751.C9JRT3.B4E1Z4	BF, BFD, CFB	PG 18 (BF, BFD, CFB)
		A0A0B4J1V1.P01780.P01767. A0A0B4J1X5.A0A0J9YVY3.P0DP02. P01762.A0A0C4DH42.P01772. P01763	IGHV3-21, IGHV3-7, IGHV3-53, IGHV3-74, IGHV7-4-1, IGHV3-30-3, IGHV3-11, IGHV3-66, IGHV3-33, IGHV3-48	PG 7 (IGHV3-11, IGHV3-74, IGHV7-4-1,…)

### Statistical analyses

#### pQTL discovery

**Fig. 5:**
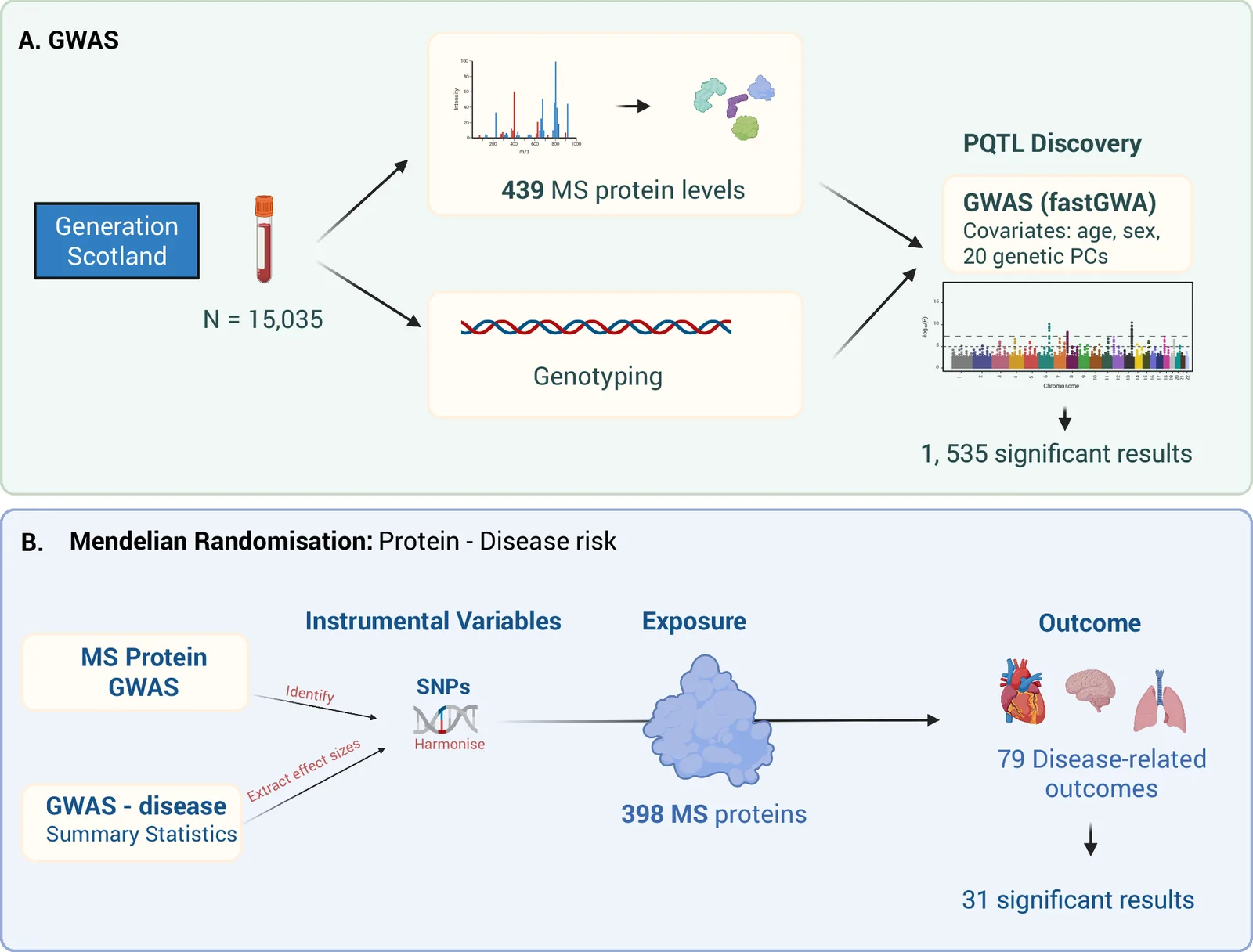
Outline of methods for GWAS and 2-sample MR. **A** GWAS: GWAS was run on 15,035 samples to identify pQTLs for 439 mass spectrometry proteins using the fastGWA option from GCTA. Age, sex and the first 20 genetic principal components were included as covariates. **B** MR: two-sample Mendelian randomisation was carried out using results from our protein GWAS and summary statistics for GWAS of the 79 selected disease outcomes. MR was run for the 398 proteins with pQTLs in the GWAS results. Figure created with BioRender.com.

GWAS were used to identify pQTLs for protein levels (*N* = 15,035, Fig. 5A). Linear regression was run using the fastGWA command in GCTA (version 1.94.1)^71^. Protein levels were transformed by rank-based inverse normalisation. Age, sex and 20 genetic principal components (to capture population structure) were included as fixed effect covariates in the GWAS models. Correlations between all protein levels and covariates are detailed in Supplementary Data 12. A sparse genetic relationship matrix (GRM) was generated using a GRM cut-off value of 0.05 and fitted as a random effect to control for family structure. A Bonferroni statistical significance threshold was set at *P* < 1.2 × 10^−10^ (genome-wide significance threshold divided by number of inferred proteins, 5 × 10^−8^/439). Cis pQTLs were defined as variants lying within 1 Mb of the transcription start site for the gene encoding the primary protein target. Lead-independent pQTLs were identified via conditional and joint analysis of the summary statistics for each protein using GCTA-COJO^71,72^. Default settings were specified, which included a stepwise selection procedure across 10 Mb regions and SNP linkage disequilibrium (LD) cut-offs set to *R*^2^ < 0.9 to avoid multicollinearity. The statistical significance threshold was once again set to *P* < 1.2 × 10^−10^. HRC^64^ imputed data from Generation Scotland was used as the reference panel for LD calculations.

### Comparison with previous pQTL studies

The discovered pQTLs were compared with those found in two previous affinity-based large-scale pQTL studies by Sun et al.^11^ and Eldjarn et al.^10^. Sun et al. used the Olink proximity extension assay for protein measurement in 54,265 UK biobank participants, while Eldjarn et al. used the SomaScan v4 assay with a cohort of 35,892 Icelanders. Additionally, we compared our results with a recent pQTL study of mass-spectrometry-based proteomics (Suhre et al.) using the Seer Proteograph nanoparticle enrichment MS platform in a discovery cohort of 1260^18^. Of the 439 proteins and protein groups quantified in our study, 58 and 75 were also included in the Sun et al. and Eldjarn et al. studies, respectively, and 114 from the discovery results in the Suhre et al. study, using “mapped UniProt ID” from their results to identify overlapping proteins. A pQTL was considered replicated if the genome-wide significant lead variant from the published studies was within 1 Mb and with an effect size in the same direction as the discovered lead variant, as well as having an LD score of >0.6.

### SNP-by-sex interactions

Sex differences in genetic effects have been described across complex traits^73^, but sex differences for molecular phenotypes remain largely underexplored. However, recently, a study of high-abundant plasma proteins measured by the same mass-spectrometry method as in our study identified modulation of the proteome by hormonal contraceptive use^74^. To explore whether there may be sex-specific effects of SNPs on protein abundance, we repeated the primary pQTL analyses using fastGWA-GE^75^ with a SNP-by-sex interaction term for all proteins. A sparse GRM was specified as per the primary pQTL analyses, and the same stringent Bonferroni correction was applied.

### Mendelian randomisation and colocalisation

To investigate possible causal associations between protein levels and disease risk, two-sample MR^76^ analyses were carried out using TwoSampleMR (version 0.6.8) for each protein with >1 independent pQTL (exposure, *n* = 398) and a list of 78 common causes of morbidity and mortality taken from Gudjonsson et al.^77^, plus critical COVID^78^ as outcomes (Fig. 4B). A full list of these outcomes can be found in Supplementary Data 6, along with source information for the summary statistics. The lead independent SNPs were included as instruments for each protein and inverse-variance weighted (IVW) MR was run alongside MR-Egger, weighted median MR, weighted mode MR and simple mode MR sensitivity analyses. Evidence of directional horizontal pleiotropy was queried for by testing for a non-zero Egger MR regression intercept. MR Steiger directionality^79^ was used to determine the causal direction of the instruments on the exposure and outcome. A Bonferroni statistical significance threshold was set at *P* < 1.59 × 10^−6^ (0.05/(number of outcomes x number of exposures)). An MR result was considered significant if the IVW *p*-value was below this threshold, at least one other method had *P* < 0.05, and it had at least 3 SNP instruments.

For each significant MR result, colocalisation analysis was performed using the R package coloc (version 5.2.3)^80^. Input regions for coloc were defined as 1 Mb ± the instrument SNP used for MR analysis. A PP.H4.abf threshold of >0.6 was used to consider a causal variant shared between the outcome and the exposure.

### Gene set and tissue enrichment

Gene set enrichment analysis and tissue-specific enrichment analysis were carried out on each of our proteins with a significant MR result using the GENE2FUNC function in the Functional Mapping and Annotation of Genome-Wide Association Studies (FUMA) online tool (version 1.8.2)^81^. Genes annotated to SNPs with *P* < 5 × 10^−8^ from the GWAS were used as input. Default settings of a minimum of two genes per gene set and an FDR Benjamini–Hochberg adjusted *P* < 0.05 were used to define significantly enriched gene sets.

For gene set enrichment analysis, gene sets from the Molecular Signatures Database (MSigDB) (hallmark, positional, curated (which includes BioCarta, KEGG and Reactome gene sets), motif, computational, gene ontology (GO), oncogenic signature, immunologic signature and cell type signature gene sets), as well as WikiPathway and GWAS catalog gene sets were used to test for enrichment. For tissue-specific enrichment analysis, the GTEx v8 54 tissue types and the GTEx v8 30 general tissue types databases were used to test for overrepresentation of differentially expressed genes in specific tissue types.

### Reporting summary

Further information on research design is available in the Nature Portfolio Reporting Summary linked to this article.

## Supplementary information

Supplementary Information

Description of Additional Supplementary Files

Supplementary Data 1

Supplementary Data 2

Supplementary Data 3

Supplementary Data 4

Supplementary Data 5

Supplementary Data 6

Supplementary Data 7

Supplementary Data 8

Supplementary Data 9

Supplementary Data 10

Supplementary Data 11

Supplementary Data 12

Reporting Summary

Transparent Peer Review file

## Source data

Source Data

## Data Availability

According to the terms of consent for Generation Scotland participants, access to data must be reviewed by the Generation Scotland Access Committee. Access requests can be made through the researcher portal (https://gsaccess.igc.ed.ac.uk) and normally take up to six weeks for approval. Further information can be found on the Generation Scotland website: https://genscot.ed.ac.uk/for-researchers/access. GWAS summary statistics are available on the Edinburgh DataShare site: https://datashare.ed.ac.uk/handle/10283/9082. Datasets used in the replication analysis can be found in the Supplementary Information of their associated publications: https://www.nature.com/articles/s41586-023-06563-x#MOESM3^10^, https://www.nature.com/articles/s41586-023-06592-6#Sec49^11^ and https://www.nature.com/articles/s41588-025-02413-w#Sec27^18^. The figure data generated in this study are provided in the Source Data file. Source data are provided with this paper.

## Code availability

All code associated with this manuscript is available open access at the following GitHub repository: https://doi.org/10.5281/zenodo.20082557^82^.
