# Supplementary material for: Genome-wide analysis of 439 mass spectrometry-based proteomic profiles in a population of 15,035 Scottish individuals
Source: Nat Commun. 2026 Sep 9;17:10659. doi: 10.1038/s41467-026-76474-8 (PMC13655163; doi:10.1038/s41467-026-76474-8)
Supplement: Supplementary file 1 [file 41467_2026_76474_MOESM15_ESM.pdf]

Corresponding author(s): Riccardo Marioni
Last updated by author(s): Sep 4, 2025

Reporting Summary

Nature Portfolio wishes to improve the reproducibility of the work that we publish. This form provides structure for consistency and transparency in reporting. For further information on Nature Portfolio policies, see our Editorial Policies and the Editorial Policy Checklist.

Statistics

For all statistical analyses, confirm that the following items are present in the figure legend, table legend, main text, or Methods section.

n/a	Confirmed	
☐	☒	The exact sample size (*n*) for each experimental group/condition, given as a discrete number and unit of measurement
☐	☒	A statement on whether measurements were taken from distinct samples or whether the same sample was measured repeatedly
☐	☒	The statistical test(s) used AND whether they are one- or two-sided<br>*Only common tests should be described solely by name; describe more complex techniques in the Methods section.*
☐	☒	A description of all covariates tested
☐	☒	A description of any assumptions or corrections, such as tests of normality and adjustment for multiple comparisons
☐	☒	A full description of the statistical parameters including central tendency (e.g. means) or other basic estimates (e.g. regression coefficient) AND variation (e.g. standard deviation) or associated estimates of uncertainty (e.g. confidence intervals)
☐	☒	For null hypothesis testing, the test statistic (e.g. *F*, *t*, *r*) with confidence intervals, effect sizes, degrees of freedom and *P* value noted<br>*Give P values as exact values whenever suitable.*
☒	☐	For Bayesian analysis, information on the choice of priors and Markov chain Monte Carlo settings
☒	☐	For hierarchical and complex designs, identification of the appropriate level for tests and full reporting of outcomes
☐	☒	Estimates of effect sizes (e.g. Cohen's *d*, Pearson's *r*), indicating how they were calculated

Our web collection on statistics for biologists contains articles on many of the points above.

Software and code

Policy information about availability of computer code

Data collection	No software was used for data collection
Data analysis	R was used for file preparation, GCTA (version 1.94.1) was used for GWAS, SNP-by-sex interaction and conditional analysis. TwoSampleMR (version 0.6.8) was used for mendelian randomisation analysis and Coloc (version 5.2.3) was used for colocalisation. FUMA (version 1.8.2) was used for gene set enrichment analysis. Code used is available open access at the following GitHub repository: https://doi.org/10.5281/zenodo.20082557

For manuscripts utilizing custom algorithms or software that are central to the research but not yet described in published literature, software must be made available to editors and reviewers. We strongly encourage code deposition in a community repository (e.g. GitHub). See the Nature Portfolio guidelines for submitting code & software for further information.

Data

Policy information about availability of data

All manuscripts must include a data availability statement. This statement should provide the following information, where applicable:

- Accession codes, unique identifiers, or web links for publicly available datasets
- A description of any restrictions on data availability
- For clinical datasets or third party data, please ensure that the statement adheres to our policy

According to the terms of consent for Generation Scotland participants, access to data must be reviewed by the Generation Scotland Access Committee.

Information on how to make an application can be found on the Generation Scotland website https://genscot.ed.ac.uk/for-researchers/access. GWAS summary statistics are available on the Edinburgh DataShare site https://datashare.ed.ac.uk/handle/10283/9082.

Research involving human participants, their data, or biological material

Policy information about studies with human participants or human data. See also policy information about sex, gender (identity/presentation), and sexual orientation and race, ethnicity and racism.

Reporting on sex and gender	Analysis included both males (N=6,581, 42%) and females (N=9,237, 58%). Sex was included as a covariate in GWAS analysis. A SNP-by-sex interaction analysis was carried out to determine any sex specific effects. Sex was self reported and confirmed using genotype data. Samples were excluded where self reported and genotype sex did not match. Preferred gender use information was not collected.
Reporting on race, ethnicity, or other socially relevant groupings	Race and ethnicity information was not included in the analysis. The cohort consists of mostly white (99%; other/missing = 1%) participants all from Scotland. 20 genetic principal components were used to account for population structure.
Population characteristics	Age, sex and 20PCs were used as covariates in the analysis. A genetic relationship matrix was also included to account for family structure. Further information on these characteristics is included in the 'Behavioural & social sciences study design' section below. No diagnoses or treatment conditions were considered in this study.
Recruitment	Individuals aged 35-65 years were randomly recruited from collaborating GP medical practices. First degree relatives (aged 18-99) of these participants were then identified and invited to participate.
Ethics oversight	All components of Generation Scotland received ethical approval from the NHS Tayside Committee on Medical Research Ethics [REC Reference Number: 05/S1401/89]. Generation Scotland has also been granted Research Tissue Bank status by the East of Scotland Research Ethics Service [REC Reference Number: 20-ES-0021], providing generic ethical approval for a wide range of uses within medical research. All participants provided written informed consent.

Note that full information on the approval of the study protocol must also be provided in the manuscript.

Field-specific reporting

Please select the one below that is the best fit for your research. If you are not sure, read the appropriate sections before making your selection.

☐ Life sciences ☒ Behavioural & social sciences ☐ Ecological, evolutionary & environmental sciences

For a reference copy of the document with all sections, see nature.com/documents/nr-reporting-summary-flat.pdf

Behavioural & social sciences study design

All studies must disclose on these points even when the disclosure is negative.

Study description	This study is a genetic study using quantitative data from the previously established Generation Scotland population cohort.
Research sample	The sample in this study consists of participants in the Generation Scotland cohort. Generation Scotland is a family based population cohort with all participants living in Scotland. The full cohort has N=23,960, with an age range of 18-99 years (mean = 47 years) and is 41% male and 59% female. There are 5,573 families with a mean size of 4 members and 1,400 participants without relatives. The subset of the cohort used for this study has N=15,035, an age range of 18-99 years (mean = 49 years) and 6,257 (42%) males and 8,778 (58%) females.
Sampling strategy	The samples used in this study came from the pre-existing Generation Scotland dataset. The sample size was determined by the number of samples with genetic, proteomic and covariate data available.
Data collection	Data collection was carried out prior to the start of this study as the data used is from an existing dataset. Participants attended an in person clinic where they filled out a pen and paper questionnaire to collect self reported phenotype data and blood samples and physical measurements were taken.
Timing	2006 to 2011
Data exclusions	Data was excluded from the analyses based on missingness. 8,925 samples were removed from the Generation Scotland cohort for this study due to either a lack of genetic, proteomic and/or covariate data.
Non-participation	All Generation Scotland participants gave broad consent to be included in all studies approved by the Generation Scotland Access Team.
Randomization	Participants have not been allocated in to experimental groups

Reporting for specific materials, systems and methods

We require information from authors about some types of materials, experimental systems and methods used in many studies. Here, indicate whether each material, system or method listed is relevant to your study. If you are not sure if a list item applies to your research, read the appropriate section before selecting a response.

Materials & experimental systems

n/a	Involved in the study
☒	☐ Antibodies
☒	☐ Eukaryotic cell lines
☒	☐ Palaeontology and archaeology
☒	☐ Animals and other organisms
☒	☐ Clinical data
☒	☐ Dual use research of concern
☒	☐ Plants

Methods

n/a	Involved in the study
☒	☐ ChIP-seq
☒	☐ Flow cytometry
☒	☐ MRI-based neuroimaging

Plants

Seed stocks

Report on the source of all seed stocks or other plant material used. If applicable, state the seed stock centre and catalogue number. If plant specimens were collected from the field, describe the collection location, date and sampling procedures.

Novel plant genotypes

Describe the methods by which all novel plant genotypes were produced. This includes those generated by transgenic approaches, gene editing, chemical/radiation-based mutagenesis and hybridization. For transgenic lines, describe the transformation method, the number of independent lines analyzed and the generation upon which experiments were performed. For gene-edited lines, describe the editor used, the endogenous sequence targeted for editing, the targeting guide RNA sequence (if applicable) and how the editor was applied.

Authentication

Describe any authentication procedures for each seed stock used or novel genotype generated. Describe any experiments used to assess the effect of a mutation and, where applicable, how potential secondary effects (e.g. second site T-DNA insertions, mosiacism, off-target gene editing) were examined.
