# Supplementary material for: Genome-wide analysis of 439 mass spectrometry-based proteomic profiles in a population of 15,035 Scottish individuals
Source: Nat Commun. 2026 Sep 9;17:10659. doi: 10.1038/s41467-026-76474-8 (PMC13655163; doi:10.1038/s41467-026-76474-8)
Supplement: Supplementary file 2 [file 41467_2026_76474_MOESM16_ESM.pdf]

nature portfolio

# Peer Review File

# Genome-wide analysis of 439 mass spectrometry-based proteomic profiles in a population of 15,035 Scottish individuals

Corresponding Author: Professor Riccardo Marioni

**This file contains all reviewer reports in order by version, followed by all author rebuttals in order by version.**

Version 0:

Reviewer comments:

Reviewer #1

(Remarks to the Author)
Richmond and co-authors employed the recently developed Scanning SWATH method to profile 439 highly abundant circulating proteins in blood samples of over 15 thousands individuals. This is one of the biggest unbiased datasets that can be used for pQTL detection. The authors also performed Mendelian association analysis to explore potential causal associations with 79 common causes of morbidity and mortality.

The authors compared their LC-MS/MS results with those generated using Olink and Somalogic platforms. The overlap, though not perfect, is very significant, proving the validity of their approach (given their more abundance-driven and unbiased approach).

The study reports several interesting findings, such as many cis- and trans-pQTLs and causal relations with morbidity/mortality, which will be interesting for the broad readership and worth follow-up studies to prove the mechanistic aspects. The study is a great resource for hyoitherses that should be checked by people interested in a specific genome locus, protein, or disease, and thus additional evidence will likely be generated by more specialized studies.

I did not identify any flaws in data analysis, interpretation, or conclusions, only some minor comments. The methodology used in handling genotype data, QTL mapping, and MR is rather routine; it meets the standards of the field. The methods section looks adequate; I only missed sections on protein and DNA preps parts. The authors provide very nice supplementary tables with detailed results for each stage of their analysis.

Minor comments:
Line 298: Hardy-Weinberg p-value <1x10ˆ6 – did you mean 10ˆ-6 here?
Usage of “PBonferoni” throughout the text – do you mean Padj here? These values are quite low, so it is important to be unambiguous whether they are adjusted or nominal.

Reviewer #2

(Remarks to the Author)
This manuscript by Richmond et al. (NCOMMS-25-70438) presents a genome-wide association study of 439 highly abundant serum protein groups derived from mass spectrometry (MS)-based proteomics in 15,035 individuals from the Generation Scotland cohort. The study reports 1,553 independent pQTLs and suggests putative causal associations between 13 proteins and 17 complex disease traits, highlighting neuropsychiatric and cardiovascular, using Mendelian Randomisation (MR). The large sample size is a significant strength, providing a valuable resource for the field of proteogenomics. However, several concerns regarding clarity, methodology, and a stronger rationale need to be addressed to enhance the rigor and interpretability of the findings. Please see my detailed comments below for concerns and suggestions.

**Major comments:**
1. Participant characteristics of the cohort are not described. A summary table of this information, either in the main

manuscript or as supplementary information, would be informative.

2. The authors highlight the advantages of MS-based proteomics integrating with genomics over previously used aptamer- or antibody-based platforms. I suggest citing an additional relevant reference (doi: 10.1038/s41588-025-02089-2) to further strengthen this point. In addition, on Page 4, Line 69, there are two references cited (16, 17). However, the focus appears to be primarily on the limited sample sizes in prior studies. I suggest revising this section to better emphasize both the strengths of MS-based proteomics over affinity assay-based approaches and the novelty of the large cohort scale presented in this study.

3. The manuscript lacks sufficient detail on the MS-based proteomics workflow, including the serum sample proteomics experiment and LC-MS acquisition parameters. Only three references (18, 61, 62) are cited without further explanation. To ensure transparency and enable proper understanding, particularly given that the raw datasets are not publicly available, a concise but complete description of the experimental procedures should be included in the manuscript.

4. The manuscript does not clearly describe the criteria or rationale used to select 439 proteins from the mass spectrometry dataset for GWAS analysis. Please clarify how these proteins were chosen, whether based on quantification reliability, data completeness, or other filtering criteria.

5. Authors briefly describe the application of within-batch drift and between-batch correction on Page 16 in Line 314-316. However, the details are insufficient to assess the robustness of mass spectrometry analysis in this large cohort. Given the large sample size of this study, rigorous quality control is essential to ensure data reliability. It is common practice to include pooled serum samples as a quality control sample to monitor instrument performance over time and/or use to correct the batch effect. Please provide evidence and methodological details to support the reliability of proteomic data.

6. The authors explain the terms “proteins” and “protein groups”, including distinctions between isoforms and related proteins within a group. However, the explanation remains unclear, and a clearer and precise explanation would be necessary. For example, Alpha-1-antitrypsin (SERPINA1), a key protein in this study, appears both as a standalone entry in Row 8 under “protein” and again across Rows 152–156 under “protein groups” in Supplementary Table 1. Why are these entries listed separately instead of being grouped under a single protein group?

7. On Page 16, Line 326, the section titled “Statistical Analyses” should be revised to provide more detailed descriptions.

8. The manuscript compares its pQTL findings with previous studies (references 9 and 10) using Olink and SomaLogic platforms, rather than validating results in an independent cohort. A relatively low overlap (~23%) in both studies is confirmed, but the implications and limitations of this low concordance are not sufficiently discussed.

9. While the authors report several interesting associations, the biological interpretation is relatively limited and occasionally overstated. The manuscript lacks supporting datasets to validate these findings. The authors are encouraged to acknowledge this limitation and suggest directions for future studies to explore the biological relevance of these associations.

**Minor comments**

The clarity and consistency of writing should be improved. Below are some specific suggestions.
1. Page 4, Line 59: “However, assay-based techniques…”. Consider revising to “However, affinity assay-based techniques…” for precision.
2. Page 11, Lines 185–186: “it is perhaps unsurprising…”. It could be revised to maintain a more formal scientific tone.
3. Page 16, Line 314: “Signals were annotated using UniProt.” This statement is unnecessary and can be removed.
4. Page 16, Lines 319–320: “107 mixed peptides where…”. Should this be “107 mixed proteins…” instead of “peptides”? Please verify for accuracy.

Version 1:

Reviewer comments:

Reviewer #1

(Remarks to the Author)
The authors have addressed the points I brought up in my previous review of the manuscript. The edited manuscript was sufficiently improved in terms of methodological details, interpretation of the results and readability. I would recommend the acceptance of the manuscript in its present form.

(Remarks on code availability)

Reviewer #2

(Remarks to the Author)

Thank you for your detailed response to my comments. The authors have thoroughly addressed the reviewer's concerns in the revised manuscript, substantially improving both its overall quality and clarity. I am satisfied with the revisions and have no further comments.

(Remarks on code availability)

**Open Access** This Peer Review File is licensed under a Creative Commons Attribution 4.0 International License, which permits use, sharing, adaptation, distribution and reproduction in any medium or format, as long as you give appropriate credit to the original author(s) and the source, provide a link to the Creative Commons license, and indicate if changes were made.
In cases where reviewers are anonymous, credit should be given to 'Anonymous Referee' and the source.
The images or other third party material in this Peer Review File are included in the article's Creative Commons license, unless indicated otherwise in a credit line to the material. If material is not included in the article's Creative Commons license and your intended use is not permitted by statutory regulation or exceeds the permitted use, you will need to obtain permission directly from the copyright holder.
To view a copy of this license, visit https://creativecommons.org/licenses/by/4.0/

We thank the reviewers for their feedback and have made major updates to improve our study in line with the suggested revisions. This includes tissue-specific expression and gene set enrichment analysis and an additional replication analysis with another mass spectrometry pQTL study. New text added to the manuscript is in red font.

**Line numbers given refer to those in the version without tracked changes.**

**REVIEWER COMMENTS**

**Reviewer #1 (Remarks to the Author):**

Richmond and co-authors employed the recently developed Scanning SWATH method to profile 439 highly abundant circulating proteins in blood samples of over 15 thousands individuals. This is one of the biggest unbiased datasets that can be used for pQTL detection. The authors also performed Mendelian association analysis to explore potential causal associations with 79 common causes of morbidity and mortality.

We would like to thank Reviewer 1 for their acknowledgement that our study utilises one of the biggest unbiased datasets that can be used for pQTL detection, as we believe this is a real strength of our study, with the sample size being an order of magnitude larger than a mass spectrometry GWAS recently published in Nature Genetics.

The authors compared their LC-MS/MS results with those generated using Olink and Somalogic platforms. The overlap, though not perfect, is very significant, proving the validity of their approach (given their more abundance-driven and unbiased approach).

We would also like to thank Reviewer 1 for highlighting that our findings within our comparative analysis using Olink and Somalogic studies are very significant despite the limited overlap of proteins between the datasets. All protein measurement methods have their limitations, and we believe that identifying consistent findings across platforms is the best way to ensure that results are replicable.

The study reports several interesting findings, such as many cis- and trans-pQTLs and causal relations with morbidity/mortality, which will be interesting for the broad readership and worth follow-up studies to prove the mechanistic aspects. The study is a great resource for hyoitherses that should

be checked by people interested in a specific genome locus, protein, or disease, and thus additional evidence will likely be generated by more specialized studies.

We thank Reviewer 1 for these thoughtful comments regarding our findings. It was our intention to generate a high impact pQTL resource that can be utilised by epidemiological and wet lab researchers interested in more specialised areas. We have made our GWAS summary statistics publicly available to ensure these findings are accessible.

I did not identify any flaws in data analysis, interpretation, or conclusions, only some minor comments. The methodology used in handling genotype data, QTL mapping, and MR is rather routine; it meets the standards of the field. The methods section looks adequate; I only missed sections on protein and DNA preps parts. The authors provide very nice supplementary tables with detailed results for each stage of their analysis.

We would like to thank Reviewer 1 for indicating areas where we could provide additional clarity in our methods.

We have added additional methodology regarding DNA preparation on **lines 407 to 412**:

*DNA extraction and Genotyping*

Samples were collected, processed and stored using standard operating procedures previously reported[62,63] and managed through a laboratory information management system (LIMS) at the Edinburgh Clinical Research Facility. A Nucleon Kit (Tepnel Life Science) with the BACC3 protocol was used for DNA extraction. DNA concentrations were determined using Picogreen and diluted to 50ng/µL for genotyping.

We have added additional methodology regarding protein preparation on **lines 428 to 444**:

High-throughput mass spectrometry proteomics was carried out using our in-house developed method on serum samples pre-processed for protein denaturation and trypsinisation[67,68].
Serum samples (5µl) were denatured in a solution of 50 µL of 8 M urea (Sigma-Aldrich, 1084870500) and 0.1 M ammonium bicarbonate (ABC, Thermo Fisher, 15645440) at pH 8.0. 5 µL of 50 mM dithiothreitol (DTT, Sigma-Aldrich, 43815) was used to reduce the proteins for 1 hour at 30 °C. The proteins were then alkylated in the dark for 30 minutes using 5 µL of 100 mM iodoacetamide (IAA,

Sigma-Aldrich, I1149). Dilution of the sample was performed with 340 µL of 0.1 M ammonium bicarbonate to a concentration of 1.5 M urea. 200 µL of the solution was treated with trypsin (12.5 µL, 0.1 µg/µL, Promega, Sequence Grade, V5117) overnight at 37 °C (at a 1/40 trypsin/total protein ratio) to digest the proteins. 25 µL of 0.1% v/v formic acid (FA, Thermo Fisher Scientific, 85178) was used to quench the digestion. C18 96-well plates (BioPureSPN Macro 96-well, 100 mg PROTO 300 C18, HNS S18V-L) were used to purify the peptides, which were eluted with 50% v/v acetonitrile (ACN, Thermo Fisher, 10001334), dried by a vacuum concentrator (Eppendorf Concentrator Plus), and redissolved in 50 µL of 0.1% v/v formic acid.

Minor comments:

Line 298: Hardy-Weinberg p-value <1x10ˆ6 – did you mean 10ˆ-6 here?

Many thanks for spotting this error. We have edited this (now line **418**) to Hardy-Weinberg p-value <1x10ˆ-6 as you suggest.

Usage of "PBonferoni" throughout the text – do you mean Padj here? These values are quite low, so it is important to be unambiguous whether they are adjusted or nominal.

Thank you for raising this lack of clarity. The usage of PBonferroni throughout the text was to represent the Bonferroni-adjusted p-value threshold, rather than a calculated p-value itself. This is described in the methods on **lines 517 – 518** (pQTL analysis) and lines **563 – 564** (MR analysis):

For the pQTL analysis: "A Bonferroni statistical significance threshold was set at P < 1.2x10-10 (genome-wide significance threshold divided by number of inferred proteins, 5x10-8/439)."

For the MR analysis: "A Bonferroni statistical significance threshold was set at P < 1.59 x 10-6 (0.05 / (number of outcomes x number of exposures))."

We have removed "Bonferroni" from the description of the threshold elsewhere in the text to avoid confusion.

**Reviewer #2 (Remarks to the Author):**

This manuscript by Richmond et al. (NCOMMS-25-70438) presents a genome-wide association study of 439 highly abundant serum protein groups derived from mass spectrometry (MS)-based proteomics in 15,035 individuals from the Generation Scotland cohort. The study reports 1,553 independent pQTLs and suggests putative causal associations between 13 proteins and 17 complex disease traits, highlighting neuropsychiatric and cardiovascular, using Mendelian Randomisation (MR). The large sample size is a significant strength, providing a valuable resource for the field of proteogenomics. However, several concerns regarding clarity, methodology, and a stronger rationale need to be addressed to enhance the rigor and interpretability of the findings. Please see my detailed comments below for concerns and suggestions.

We thank reviewer 2 for taking the time to review our manuscript and provide feedback to help us improve our paper.

**Major comments:**

1. Participant characteristics of the cohort are not described. A summary table of this information, either in the main manuscript or as supplementary information, would be informative.

We have now added a table describing the demographics of the cohort in Supplementary table 1. This is referred to on **line 405**:

"The demographics of the cohort can be observed in Supplementary Table 1".

2. The authors highlight the advantages of MS-based proteomics integrating with genomics over previously used aptamer- or antibody-based platforms. I suggest citing an additional relevant reference (doi: 10.1038/s41588-025-02089-2) to further strengthen this point. In addition, on Page 4, Line 69, there are two references cited (16, 17).
However, the focus appears to be primarily on the limited sample sizes in prior studies. I suggest revising this section to better emphasize both the strengths of MS-based proteomics over affinity assay-based approaches and the novelty of the large cohort scale presented in this study.

We thank reviewer two for their suggestion of an additional reference to add to strength our point and have now added this on **line 71**.

We have also revised our introduction to better emphasise the strengths of MS-based proteomics over affinity-based approaches on **lines 54 to 69**:

To date, large-scale studies have used targeted aptamer- or affinity-based platforms, that can assess upwards of 3,000 (Olink Explore 3,072 panel) and 7,000 pre-defined proteins (SomaLogic SomaScan assay)[7–9]. Genome wide association studies (GWAS) of these proteins have helped to give mechanistic insights into multiple disease outcomes – including those where blood is not the primary tissue of interest[10–13]. However, affinity assay-based techniques have limitations. Quantification of functionally important, high abundance serum proteins can be inaccurate due to signals exceeding the dynamic range of the array and the presence of different isoforms[14]. They are also limited by a pre-defined list of protein targets, off target binding and interference caused by autoantibodies[15], as well as lack of concordance across arrays[16]. In contrast, untargeted approaches for proteomics such as mass spectrometry are unbiased and yield comprehensive and granular coverage of the high-abundance proteome. This non-reliance on epitope confers higher analytical specificity relative to affinity-based methods and enables direct interrogation of protein isoforms and peptide-level variation. When integrated with genomic data, this resolution facilitates direct mapping of genetic variation to specific peptides and proteins, offering a deeper mechanistic view of genotype-protein relationships.

We also added additional text to emphasise the novelty of our large sample size on lines **69** to **73:**

To date, only a small number of studies have performed GWAS on mass spectrometry-measured proteins. However, their sample sizes have been limited to under 3,000 individuals[9,17,18]. This is an important limitation as GWAS typically require large sample sizes to achieve adequate statistical power to detect variants with small effect sizes.

and lines **82 – 84**:

Here, we characterise the genetic architecture of 439 mass spectrometry-measured proteins in 15,035 individuals from the Generation Scotland study. This study is 5-10 times larger than recently published GWAS of mass spectrometry proteomics[9,17,18].

3. The manuscript lacks sufficient detail on the MS-based proteomics workflow, including the serum sample proteomics experiment and LC-MS acquisition parameters. Only three references (18, 61, 62) are cited without further explanation. To ensure transparency and enable proper understanding, particularly given that the raw datasets are not publicly available, a concise but complete description of the experimental procedures should be included in the manuscript.

We would like to thank reviewer 2 for suggesting improvements to our LC-MS methodology to ensure transparency and proper understanding. We have revised our LC-MS methods section in accordance with this feedback:

Lines **428 - 465**

High-throughput mass spectrometry proteomics was carried out using our in-house developed method on serum samples pre-processed for protein denaturation and trypsinisation[67,68]. Serum samples (5µl) were denatured in a solution of 50 µL of 8 M urea (Sigma-Aldrich, 1084870500) and 0.1 M ammonium bicarbonate (ABC, Thermo Fisher, 15645440) at pH 8.0. 5 µL of 50 mM dithiothreitol (DTT, Sigma-Aldrich, 43815) was used to reduce the proteins for 1 hour at 30 °C. The proteins were then alkylated in the dark for 30 minutes using 5 µL of 100 mM iodoacetamide (IAA, Sigma-Aldrich, I1149). Dilution of the sample was performed with 340 µL of 0.1 M ammonium bicarbonate to a concentration of 1.5 M urea. 200 µL of the solution was treated with trypsin (12.5 µL, 0.1 µg/µL, Promega, Sequence Grade, V5117) overnight at 37 °C (at a 1/40 trypsin/total protein ratio) to digest the proteins. 25 µL of 0.1% v/v formic acid (FA, Thermo Fisher Scientific, 85178) was used to quench the digestion. C18 96-well plates (BioPureSPN Macro 96-well, 100 mg PROTO 300 C18, HNS S18V-L) were used to purify the peptides, which were eluted with 50% v/v acetonitrile (ACN, Thermo Fisher, 10001334), dried by a vacuum concentrator (Eppendorf Concentrator Plus), and redissolved in 50 µL of 0.1% v/v formic acid.

Liquid chromatography-mass spectrometry (LC-MS) was carried out on the processed samples using the Agilent 1290 Infinity II system (LC) and a Triple TOF 6600 mass spectrometer (SCIEX)[19]. A Phenomenex Luna Omega LC Column (1.6u PS C18 100A, 30 x 2.1 mm) was used to separate 2µg of peptides over a 3-minute gradient. Buffer A (0.1% v/v FA) and Buffer B (ACN/0.1% v/v FA) were used to create a linear gradient, starting from 1% Buffer B and reaching 40% Buffer B over 3 minutes with an 800 µL/min flow rate. At a 40% gradient, washing and re-equilibration steps were carried out in the following manner: Buffer B was increased from 40% to 80% over 0.5 min, followed by 80% Buffer

B for 0.2 min, and then decreased from 80% to 3% Buffer B over 0.1 min. Re-equilibration at 3% Buffer B was maintained for 1 min until the next sample injection.

Sample acquisition was carried out using a Scanning SWATH method[19]. High-sensitivity mode was used for the mass spectrometer. The source conditions were as follows: source gas one at 15 psi, source gas two at 20 psi, curtain gas at 25 psi, temperature at 0 °C, IonSpray floating voltage at 5,500 V, and declustering potential at 80 V. Rolling collision energies were determined using the following equation: CE=0.034×□/□+2, where m/z represents the centre of the scanning quadrupole bin. The Scanning SWATH parameters were as follows: total cycle time 0.69 s, transmission window width 10 Da, Sample duration 3.103 min, precursor range 450-850 Da, fragment range 100-1500 Da and effective accumulation time 16.90 ms.

4. The manuscript does not clearly describe the criteria or rationale used to select 439 proteins from the mass spectrometry dataset for GWAS analysis. Please clarify how these proteins were chosen, whether based on quantification reliability, data completeness, or other filtering criteria.

We have added the following information on lines **467 - 473** to clarify the quality control steps that led to 439 proteins in the mass spectrometry dataset:

DIA-NN (version 1.8.12)[69] in Robust LC (high precision) was used to process the raw data using default parameters. A human plasma project-independent public spectral library[70] was used for identification and refined with parameters estimated from the data being processed[67]. Precursor false discovery rate (FDR) filtering was set to 1%. Additional processing was carried out in R. A gene group q-value filter of 1% was applied. Samples with a minimum precursor identification of 2000 were retained in the analysis, and 80% prevalence rate for precursors was used to filter QC samples.

5. Authors briefly describe the application of within-batch drift and between-batch correction on Page 16 in Line 314-316. However, the details are insufficient to assess the robustness of mass spectrometry analysis in this large cohort. Given the large sample size of this study, rigorous quality control is essential to ensure data reliability. It is common practice to include pooled serum samples as a quality control sample to monitor instrument performance over time and/or use to correct the batch effect. Please provide evidence and methodological details to support the reliability of proteomic data.

We have added methodological details to support the reliability of the proteomic data on lines **473 - 476**:

A cubic-spline model was fit using the repeat injections of pooled QC samples and used to correct samples for within-batch drift[71]. Between-batch correction was performed using limma v3.54.2 linear batch-correction algorithm[72].

6. The authors explain the terms "proteins" and "protein groups", including distinctions between isoforms and related proteins within a group. However, the explanation remains unclear, and a clearer and precise explanation would be necessary. For example, Alpha-1-antitrypsin (SERPINA1), a key protein in this study, appears both as a standalone entry in Row 8 under "protein" and again across Rows 152–156 under "protein groups" in Supplementary Table 1. Why are these entries listed separately instead of being grouped under a single protein group?

We have added additional information and an exemplar annotation table to improve clarity of the distinctions between proteins and protein groups on **lines 478 to 505**:

439 proteins and protein groups were considered, 133 (30.3%) of these were uniquely mapped to one UniProt ID (termed "proteins"), with the remaining 306 (69.7%) mapping to multiple potential UniProt IDs (termed "protein groups"). 199 of these protein groups represent different isoforms of the same protein, and 107 mixed protein groups where the signal mapped to different proteins. 138 out of the 199 isoform signals were mapped to proteins that were identified more than once (repeated isoform groups). We use a "GX" naming system, where "G" signifies a group and "X" refers to a number to identify these repeated isoform groups (**Table 1**). For example, Apolipoprotein D was identified twice, with one of the signals annotating to three isoforms [UniProt identifiers: P05090, C9JF17, C9JX71] and was given the name Apolipoprotein D (G1) and another signal mapped to two isoforms [UniProt identifiers: P05090.C9JF17], therefore was named Apolipoprotein D (G2). 61 signals were annotated to protein isoform groups that did not repeat; these therefore only have a "G" after their protein name. We used a "PG X" naming system to identify the 107 signals that map to multiple different proteins. For example, one signal is annotated to multiple immunoglobulins (Immunoglobulin lambda variable 3-27, Immunoglobulin lambda variable 3-25 and Immunoglobulin lambda variable 3-16), and therefore is referred to as protein group 1 (PG 1). As can be observed with the PG1 example, in many cases, the protein groups with mixed labels contain related proteins. In some instances, a protein is identified as a unique signal and as several protein groups. For example, one mass spectrometry signal mapped to Alpha-1-antitrypsin identified via a single UniProt

ID P01009, therefore was named Alpha-1-antitrypsin. However, five other mass spectrometry signals mapped to five different combinations of UniProt IDs that were all related to Alpha-1-antitrypsin and were therefore named Alpha-1-antitrypsin G1 to G5. Given that these groups originate from different signals, they were maintained as separate entities in downstream analysis. An annotation of the 439 proteins considered in this study is provided in **Supplementary Table 2.**

**Table 2: Illustration of protein annotation strategy.**

| ***Protein Category*** | ***Count (n)*** | ***Example UniProtID(s)*** | ***Gene Symbol(s)*** | ***Final annotation*** |
|---|---|---|---|---|
| ***Unique Proteins*** | 133 | P00450 | CP | Ceruloplasmin |
| ***Gene-Derived Protein Groups (G)*** | 199 | P02647.F8W696 | APOA1, APOA1 | Apolipoprotein A-I (G) |
| | | P00736.F5H2D0.B4DPQ0 | C1R, C1R, C1R | Complement C1r subcomponent (G1) |
| | | P00736.F5H1V0.F5H6Y3.B4DPQ0 | C1R, C1R, C1R, C1R | Complement C1r subcomponent (G2) |
| ***Mixed Protein Groups (PG)*** | 107 | P80748.A0A075B6K5 | IGLV3-21, IGLV3-9 | PG 70 (IGLV3-21, IGLV3-9) |
| | | P00751.C9JRT3.B4E1Z4 | BF, BFD, CFB | PG 18 (BF, BFD, CFB) |

| | | | |
|---|---|---|---|
| | A0A0B4J1V1.P01780.P01767.<br>A0A0B4J1X5.A0A0J9YVY3.P0DP02.<br>P01762.A0A0C4DH42.P01772.<br>P01763 | IGHV3-21,<br>IGHV3-7, IGHV3-53,<br>IGHV3-74,<br>IGHV7-4-1,<br>IGHV3-30-3,<br>IGHV3-11,<br>IGHV3-66,<br>IGHV3-33,<br>IGHV3-48 | PG 7<br>(IGHV3-11,<br>IGHV3-74,<br>IGHV7-4-1, ...) |

7. On Page 16, Line 326, the section titled "Statistical Analyses" should be revised to provide more detailed descriptions.

Thank you for this comment, we believe the heading structure may have been unclear. The Statistical Analyses section incorporates the five subsequent sections, which each include detailed descriptions of the relevant analysis. The sentences immediately following "Statistical Analyses" have been moved to the section describing the cohort as these include broad, general points about the input data for each of these analyses. They are now situated on lines **403 – 405**.

8. The manuscript compares its pQTL findings with previous studies (references 9 and 10) using Olink and SomaLogic platforms, rather than validating results in an independent cohort. A relatively low overlap (~23%) in both studies is confirmed, but the implications and limitations of this low concordance are not sufficiently discussed.

Thank you for highlighting an area where we could provide more discussion.
We have added additional comparative analysis with another mass-spectrometry pQTL study and expanded our discussion of the interpretation of this overlap. Together with our earlier comparison to affinity-based studies, this new analysis situates our findings within the context of three published studies. We have also added a new figure which emphasises the replication of our results across the three studies. We would like to emphasise that this analysis illustrates that several of our findings replicate across cohorts regardless of measurement platform used. As the Eliptica platform is

currently being run on 50,000 volunteers from the UK Biobank study, we will be able to run direct comparisons in the future.

On **lines 528 to 540** we describe the replication analysis methodology:

The discovered pQTLs were compared with those found in two previous affinity-based large-scale pQTL studies by Sun *et al*[11] and Eldjarn *et al*[10]. Sun *et al* used the Olink proximity extension assay for protein measurement in 54,265 UK biobank participants, while Eldjarn *et al* used the SomaScan v4 assay with a cohort of 35,892 Icelanders. Additionally, we compared our results with a recent pQTL study of mass-spectrometry-based proteomics (Suhre et. al.,) using the Seer Proteograph nanoparticle enrichment MS platform in a discovery cohort of 1,260[18]. Of the 439 proteins and protein groups quantified in our study, 58 and 75 were also included in the Sun *et al* and Eldjarn *et al* studies respectively, and 114 from the discovery results in the Suhre *et al* study, using "mapped UniProt ID" from their results to identify overlapping proteins. A pQTL was considered replicated if the genome-wide significant lead variant from the published studies was within 1Mb and with an effect size in the same direction as the discovered lead variant, as well as having an LD score of >0.6.

On **lines 132 to 143** we describe the replication analysis results:

The significant pQTLs discovered in our study were compared to three previous proteomics GWASs. These include two large-scale affinity-based proteomics GWASs using Olink Explore 3072 PEA in the UK Biobank[11] and SomaScan v4 in the deCODE study[10], and a smaller scale mass-spectrometry proteomics GWAS using the MS-based Seer Proteograph platform in the Tarkin study[18]. For all significant pQTLs in the current study, we performed lookup analyses in these studies, where the same protein was assessed and also demonstrated significant associations. We replicated 46 of 199 (23.12%), 64 of 275 (23.27%) and 10 of 25 (40%) pQTLs with the Sun et al[11]., Eldjarn et al[10]., and Suhre et. al[18]., studies respectively (see **Table 1**). Effect sizes of the overlapping associations correlated highly between platforms ($R_{Pearson}$ = 0.94 (MS:UKB[11]); 0.90 (MS:deCODE cohort[10]); 0.89 (MS:Tarkin[18]), (**Figure 2.**). A list of the overlapping proteins-SNP associations can be found in **Supplementary Tables 7, 8 and 9**.

After **line 145**, a table describing our overlapping findings with three studies can be observed:

| **Comparator GWAS study** | **Sun et. al.,**[11] | **Eldjarn et. al.,**[10] | **Suhre et. al.,**[18] |
|---|---|---|---|
| **Cohort** | UK Biobank | Decode | Tarkin |

| | | | |
|---|---|---|---|
| **Sample Size** | 54,219 | 35,892 | 1,260 |
| **Proteomics Platform** | Olink | SomaScan | Seer |
| **N proteins tested** | 2,923 | 4,719 | 1,980 |
| **Overlap of proteins*** | 58 | 75 | 114 |
| **Overlap of proteins with significant associations*** | 51 | 69 | 22 |
| **N significant associations with overlapping proteins in comparator study** | 199 | 275 | 25 |
| **Replicated pQTLs (%)*** | 46 (23.1%) | 64 (23.3%) | 10 (40%) |

**Table 1. Comparison of discovery pQTLs with three comparator studies.** The three comparator studies considered include two large-scale affinity-based proteomics GWASs using Olink Explore 3072 PEA in the UK Biobank[11] and SomaScan v4 in the deCODE study[10], and a smaller scale mass-spectrometry proteomics GWAS using the MS-based Seer Proteograph platform in the Tarkin study[18]. *These rows refer to overlap between our study (Eliptica MS GWAS) and the respective study for each column.

Visual comparison of effect sizes for replicating findings in the other three studies can be observed on **line 155**:

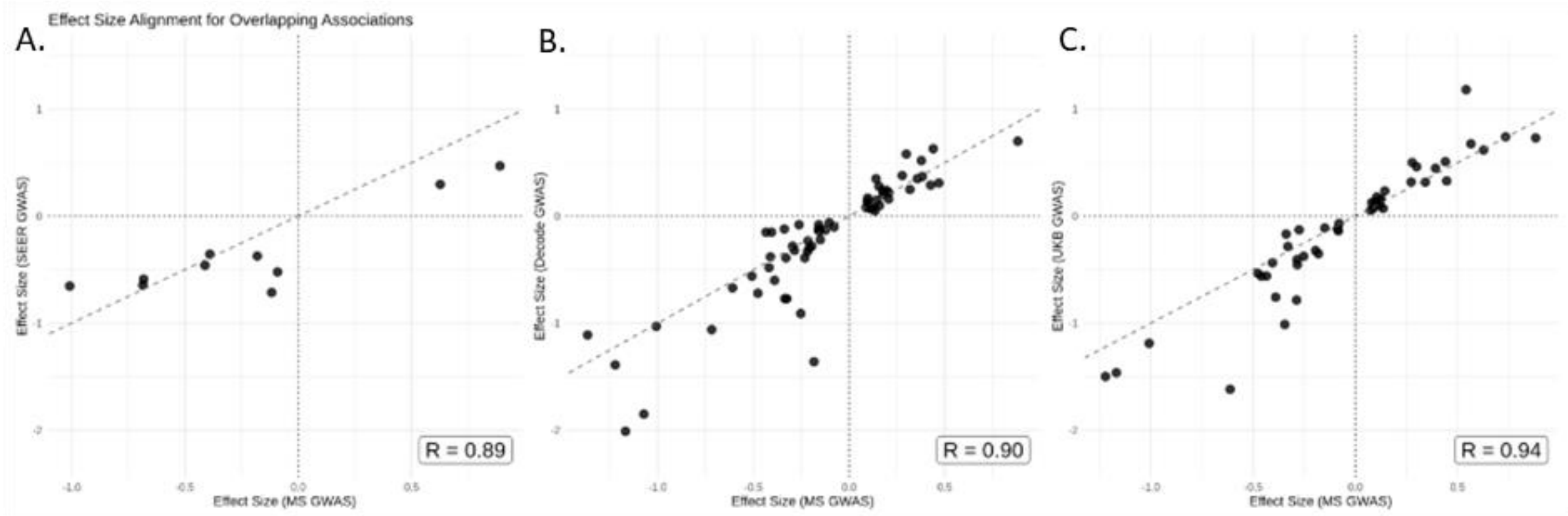


**Figure. 2. Overlapping associations with comparator pQTL studies.** Overlapping **a**ssociations were defined as those where the genome-wide significant lead variant from the comparator study was within 1Mb of the discovered lead variant, with a same direction effect size, and has an LD score of >0.6. "MS GWAS" refers to the current study. R denotes Pearson R, calculated using cor.test from the Statistics package in R. **A.** 10 overlapping pQTLs with the Seer proteomics GWAS (Suhre et. al.,[18]); **B.** 64 overlapping pQTLs with the Icelandic SomaScan v4 platform GWAS (Eldjarn et. al.,[10]); **C.** 46 overlapping pQTLs with the UK Biobank GWAS (Sun et. al.,[11]).

9. While the authors report several interesting associations, the biological interpretation is relatively limited and occasionally overstated. The manuscript lacks supporting datasets to validate these findings. The authors are encouraged to acknowledge this limitation and suggest directions for future studies to explore the biological relevance of these associations.

Thank you for highlighting that we have identified several interesting results. We have taken additional steps to address your other comments. In terms of the biological interpretation, we have additionally run Gene Set and Tissue enrichment analysis and colocalisation analysis on the MR results. We have added text to our discussion to acknowledge the limitations to our work and to suggest future studies.

The methods for the colocalisation analysis are described in lines **568 to 571** and Gene Set and Tissue Enrichment on lines **573 to 587**:

For each significant MR result, colocalisation analysis was performed using the R package coloc[82]. Input regions for coloc were defined as 1Mb +/- the instrument SNP used for MR analysis. A PP.H4.abf threshold of >0.6 was used to consider a causal variant shared between the outcome and the exposure.

**Gene Set and Tissue Enrichment**

Gene set enrichment analysis and tissue specific enrichment analysis were carried out on each of our proteins with a significant MR result using the GENE2FUNC function in the Functional Mapping and Annotation of Genome-Wide Association Studies (FUMA) online tool[83]. Genes annotated to SNPs with $P < 5 \times 10^{-8}$ from the GWAS were used as input. Default settings of a minimum of two genes per gene set and an FDR Benjamini-Hochberg adjusted $P < 0.05$ were used to define significantly enriched gene sets.

For gene set enrichment analysis, gene sets from the Molecular Signatures Database (MSigDB) (hallmark, positional, curated (which includes BioCarta, KEGG and Reactome gene sets), motif, computational, gene ontology (GO), oncogenic signature, immunologic signature and cell type signature gene sets), as well as WikiPathway and GWAS catalog gene sets were used to test for

enrichment. For tissue specific enrichment analysis, the GTEx v8 54 tissue types and the GTEx v8 30 general tissue types databases were used to test for overrepresentation of differentially expressed genes (DEGs) in specific tissue types.

We report the results of the colocalisation analysis in lines **189 - 193** with results added to Supplementary Table 10:

To further support our findings of causal associations between our protein exposures and disease outcomes we ran colocalisation analysis using coloc. 17 out of the 31 (54.8%) significant MR results showed evidence (PP.H4.abf>0.6) of a shared causal variant between the exposure and outcome (Supplementary Table 10).

On **lines 210 to 236**: we describe the results of the gene set and tissue enrichment analyses:

*Gene set and tissue enrichment analysis*

To further understand the biological roles of each protein with significant MR result~~s~~, we ran gene set enrichment analysis using FUMA. The full list of gene set databases used to test for enrichment can be found in the methods. All 17 proteins were found to have significant ($P < 0.05$) enrichment (**Supplementary Table 11)**. Pathways related to lipids and lipoproteins saw the most enrichment, with 144 pathways, from 10 gene set databases, enriched across 11 of the proteins. Many pathways related to specific diseases in the GWAS Catalog geneset database were found to be enriched, with the most abundant being those related to schizophrenia and age related macular degeneration (AMD), with enrichment across 7 proteins, followed by Alzheimer's disease and ASD whose related pathways were both enriched in 5 proteins.

Genes associated with ITIH4 and ITIH3, two of our proteins with significant MR results with psychiatric conditions, were significantly enriched in pathways related to the psychiatric conditions ASD, Schizophrenia, Bipolar Disorder and Depression. They were also both significantly enriched in brain related pathways including brain morphology, subcortical volume and cortical surface area. Genes associated with IGHM and APOM, proteins with MR associations with Alzheimer's disease and neutrophil count respectively, were both significantly enriched in immunology related pathways, including interferon signalling, autoimmune thyroid disease and MHC class II protein complex pathways.

Nine proteins were found to be significantly (P < 0.05) overrepresented in at least one tissue type (**Supplementary Table 12**). Genes associated with IGHM were found to be significantly overrepresented in blood, lung and spleen tissue and underrepresented in ovarian tissue and genes associated with SERPINA1, a protein with an MR association with ASD, were found to be overrepresented in liver tissue.

Discussion of the colocalisation results, gene set and tissue enrichment analyses has been incorporated into several sections of the discussion:

Lines **263** – **265**

Accordingly, significant GWAS loci associated with AAT were found to be overrepresented in liver tissue, on tissue-enrichment analysis.

Lines **282** -**285**

Gene set enrichment analysis of APOE-associated loci demonstrates enrichment in metabolic pathways and, via the GWAS Catalog geneset database, diseases such as Alzheimer's Disease, non-alcoholic fatty liver disease and metabolic disorders.

Lines **295 - 306**

Further, gene set enrichment analysis for the significant GWAS loci in ITIH4 and ITIH3 identified significant enrichment in pathways related to psychiatric disorders, including ASD, Schizophrenia Bipolar Disorder and Depression. This also identified significant enrichment in pathways related to brain morphology, subcortical volume and cortical surface area, aligning with well-described structural brain changes in Schizophrenia[35] and ASD[36]. Results for neither protein demonstrated tissue-specific enrichment. Our results build on existing evidence of a role of inter-alpha-trypsins in neuropsychiatric disorders from both genetic and proteomic studies. Schizophrenia is a highly heritable condition[37] and previous large-scale GWAS studies have identified SNPs in *ITIH3/4* in association with Schizophrenia[38–40] and also between *SERPINA1* and ASD[41] and *ITIH3/4* and ASD[42]. However, colocalisation analysis (PP(H3) = >0.99), did not support the same causal variant for ITIH3 and Schizophrenia, ITIH4 and ASD or AAT and ASD.

Lines **312** – **314**

However, although our results suggest that increased AAT could be protective against ASD, colocalisation analysis revealed a posterior probability (PP) of H3 as >0.99, implying no shared causal variant.

Lines **335** - **349**

Gene set enrichment analysis for the significant GWAS loci associated with this protein identified enrichment in pathways related to interferon signalling, increasingly implicated in Alzheimer disease[47], and also pathways related to autoimmune thyroid disease and MHC class II protein complexes. Further, the IGHM-associated genes were significantly enriched in blood, lung and spleen, aligning with immunological functions. The immune system is thought to play a role in the progression of AD[48,49] and decreased levels of IgM have been found in patients with AD than with age and sex matched healthy controls ($n_{cases}$ = 26, $n_{controls}$ = 26; $n_{cases}$ = 30, $n_{controls}$ = 30)[50,51]. However, findings on this topic have been inconsistent as IGHM has previously been shown to be a potential biomarker for AD, with increased levels found in patients with AD than with healthy controls ($n_{cases}$ = 45, $n_{controls}$ = 45)[52]. IgM levels in frontal cortex tissue have also been found to be increased in people with late-stage (stages V and VI) AD compared to healthy controls of the same age ($n_{cases}$ = 11, $n_{controls}$ = 9)[52,53]. Further, colocalisation analysis revealed a posterior probability of >0.99 for H3, suggesting that while the both the disease and protein have causal variants in the region, they do not share a causal variant.

We added text to the discussion to acknowledge the limitation of no replication, and to suggest future work on lines **368 to 377**:

Our replication analysis included comparison with three independent studies using three different proteomics technology platforms demonstrated 23-40% replication. Nonetheless, we acknowledge that replication in an external cohort with proteomic data generated using the same methodology would be desirable. Additionally, our study and comparative analysis included individuals of European ancestry only. Future work should focus on generating mass spectrometry datasets and replicating findings in non-European cohorts. This study provides a valuable pQTL resource and suggests potential causal relationships between proteins and health conditions. Follow-up studies will be required to determine the underlying mechanisms of these relationships and to confirm their validity.

Finally, the conclusion has been updated to reflect these results (lines **384 – 386**):

MR analysis revealed 13 proteins that have potential causal associations with 17 disease traits, though the presence of shared causal variants was only supported by subsequent colocalization analysis for 17 of the 31 significant MR results.

**Minor comments**

The clarity and consistency of writing should be improved. Below are some specific suggestions.

1. Page 4, Line 59: “However, assay-based techniques…”. Consider revising to “However, affinity assay-based techniques…” for precision.

This error has now been corrected on line **58**.

2. Page 11, Lines 185–186: “it is perhaps unsurprising…”. It could be revised to maintain

We have now updated this sentence, now on lines **267-269**: Given its involvement in a diverse range of pathways, it is biologically plausible that pQTLs within *SERPINA1* have wide-ranging associations.

3. Page 16, Line 314: “Signals were annotated using UniProt.” This statement is unnecessary and can be removed.

We have deleted this sentence from the manuscript.

4. Page 16, Lines 319–320: “107 mixed peptides where…”. Should this be “107 mixed proteins…” instead of “peptides”? Please verify for accuracy.

Thank you for highlighting this lack of clarity. We have updated this section to describe these as “107 mixed protein groups” as well as providing additional explanation on how the proteins were annotated on lines **480 – 505**:

199 of these protein groups represent different isoforms of the same protein, and 107 mixed protein groups where the signal mapped to different proteins. 138 out of the 199 isoform signals were mapped to proteins that were identified more than once (repeated isoform groups). We use a “GX” naming system, where “G” signifies a group and “X” refers to a number to identify these repeated

isoform groups (**Table 1**). For example, Apolipoprotein D was identified twice, with one of the signals annotating to three isoforms [UniProt identifiers: P05090, C9JF17, C9JX71] and was given the name Apolipoprotein D (G1) and another signal mapped to two isoforms [UniProt identifiers: P05090.C9JF17], therefore was named Apolipoprotein D (G2). 61 signals were annotated to protein isoform groups that did not repeat; these therefore only have a "G" after their protein name. We used a "PG X" naming system to identify the 107 signals that map to multiple different proteins. For example, one signal is annotated to multiple immunoglobulins (**Immunoglobulin lambda variable 3-27, Immunoglobulin lambda variable 3-25 and Immunoglobulin lambda variable 3-16), and therefore is referred to as protein group 1 (PG 1). As can be observed with the PG1 example,** in many cases, the protein groups with mixed labels contain related proteins. In some instances, a protein is identified as a unique signal and as several protein groups. For example, one mass spectrometry signal mapped to Alpha-1-antitrypsin identified via a single UniProt ID P01009, therefore was named Alpha-1-antitrypsin. However, five other mass spectrometry signals mapped to five different combinations of UniProt IDs that were all related to Alpha-1-antitrypsin and were therefore named Alpha-1-antitrypsin G1 to G5. Given that these groups originate from different signals, they were maintained as separate entities in downstream analysis.
