# Supplementary material for: Genome-wide analysis of 439 mass spectrometry-based proteomic profiles in a population of 15,035 Scottish individuals
Source: Nat Commun. 2026 Sep 9;17:10659. doi: 10.1038/s41467-026-76474-8 (PMC13655163; doi:10.1038/s41467-026-76474-8)
Supplement: Supplementary file 3 [file 41467_2026_76474_MOESM1_ESM.pdf]

Genome-wide analysis of 439 mass spectrometry-based proteomic profiles in a population of 15,035 Scottish individuals

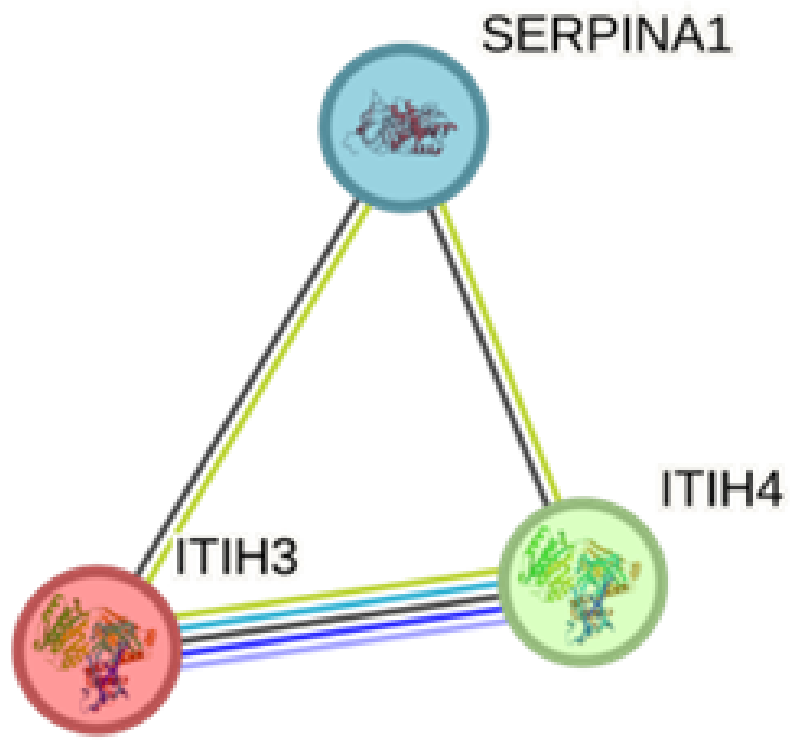

Supplementary Figure 1. StringDB[1] network of three proteins demonstrating putative causal associations with neuropsychiatric disorders. SERPINA1 (alpha-1-antitrypsin) and ITIH4 (inter-alpha-trypsin inhibitor heavy chain 4) associated with autism-spectrum disorder. ITIH3 (inter-alpha-trypsin inhibitor heavy chain 3) associated with schizophrenia. Default StringDB settings were used: minimum required interaction score = 0.4, no limit to maximum number of interactors, edges represent protein-protein associations, light green = textmining, black = co-expression, light purple = protein homology, dark blue = gene co-occurrence, green = gene-neighbourhood. Download date: 05/03/2025.
