# Supplementary material for: Genome-wide analysis of 439 mass spectrometry-based proteomic profiles in a population of 15,035 Scottish individuals
Source: Nat Commun. 2026 Sep 9;17:10659. doi: 10.1038/s41467-026-76474-8 (PMC13655163; doi:10.1038/s41467-026-76474-8)
Supplement: Supplementary file 4 [file 41467_2026_76474_MOESM2_ESM.docx]

File Name: Supplementary Data 1

Description: Genome wide significant independent pQTL associations discovered from COJO analysis. Tests run are two-sided regression models. Gene annotations for pQTL rsid and protein trait added from ensembl

File Name: Supplementary Data 2

Description: Genome wide significant results of SNP-by-sex interaction GWAS analysis

File Name: Supplementary Data 3

Description: Replicated protein-SNP associations found between discovery cohort and Sun et al replication cohort. An association was considered to be replicated if the genome wide significant lead variant from Sun et al was within 1Mb of the discovered lead variant, the effect size was in the same direction and there was an LD score (R2) of > 0.6 between the variants

File Name: Supplementary Data 4

Description: Replicated protein-SNP associations found between discovery cohort and Eldjarn et al replication cohort. An association was considered to be replicated if the genome wide significant lead variant from Eldjarn et al was within 1Mb of the discovered lead variant, the effect size was in the same direction and there was an LD score (R2) of > 0.6 between the variants

File Name: Supplementary Data 5

Description: Replicated protein-SNP associations found between discovery cohort and Suhre et al replication cohort. An association was considered to be replicated if the genome wide significant lead variant from Suhre et al was within 1Mb of the discovered lead variant, the effect size was in the same direction and there was an LD score (R2) of > 0.6 between the variants

File Name: Supplementary Data 6

Description: Full list and source information of the 79 common causes of morbidity and mortality used as outcomes in the MR analysis

File Name: Supplementary Data 7

Description: Significant protein-trait MR associations and colocalisation posterior probabilities. Output from inverse variance weighted (IVW), MR-Egger, weighted median, weighted mode and simple mode two-sample MR methods are shown. Significance was determined using IVW method. Evidence of directional horizontal pleiotropy was queried for by testing for a non-zero Egger MR regression intercept. MR Steiger directionality was used to determine the causal direction of the instruments on the exposure and outcome.

File Name: Supplementary Data 8

Description: Significant associations found from two-sided hypergeometric test gene set enrichment analysis in FUMA. Default settings of the GENE2FUNC function in FUMA of N_overlap >= 2 and adjP < 0.05 were used to define significance

File Name: Supplementary Data 9

Description: Significant associations found from two-sided hypergeometric test tissue specific enrichment analysis in FUMA. Default settings of the GENE2FUNC function in FUMA of N_overlap >= 2 and adjP < 0.05 were used to define significanc

File Name: Supplementary Data 10

Description: Demographics of the Generation Scotland cohort

File Name: Supplementary Data 11

Description: Protein name annotation from UniProt for the 439 proteins quantified by mass spectrometry

File Name: Supplementary Data 12

Description: Two-tailed Pearson correlations between all protein levels and covariates
